# Unlocking the potential of T‐cell metabolism reprogramming: Advancing single‐cell approaches for precision immunotherapy in tumour immunity

**DOI:** 10.1002/ctm2.1620

**Published:** 2024-03-11

**Authors:** Lihaoyun Huang, Haitao Li, Cangang Zhang, Quan Chen, Zaoqu Liu, Jian Zhang, Peng Luo, Ting Wei

**Affiliations:** ^1^ Department of Oncology Zhujiang Hospital Southern Medical University Guangzhou China; ^2^ The First Clinical Medical School Southern Medical University Guangzhou China; ^3^ Department of Oncology Taishan People's Hospital Guangzhou China; ^4^ Department of Pathogenic Microbiology and Immunology School of Basic Medical Sciences Xi'an Jiaotong University Xi'an Shaanxi China; ^5^ Department of Neurosurgery Xiangya Hospital Central South University Changsha Hunan China; ^6^ Key Laboratory of Proteomics Beijing Proteome Research Center National Center for Protein Sciences (Beijing) Beijing Institute of Lifeomics Beijing China; ^7^ Key Laboratory of Medical Molecular Biology Chinese Academy of Medical Sciences Department of Pathophysiology Peking Union Medical College Institute of Basic Medical Sciences Beijing China

**Keywords:** immunotherapy, metabolic reprogramming, single‐cell RNA sequencing, T‐cell metabolism, tumour immunity

## Abstract

As single‐cell RNA sequencing enables the detailed clustering of T‐cell subpopulations and facilitates the analysis of T‐cell metabolic states and metabolite dynamics, it has gained prominence as the preferred tool for understanding heterogeneous cellular metabolism. Furthermore, the synergistic or inhibitory effects of various metabolic pathways within T cells in the tumour microenvironment are coordinated, and increased activity of specific metabolic pathways generally corresponds to increased functional activity, leading to diverse T‐cell behaviours related to the effects of tumour immune cells, which shows the potential of tumour‐specific T cells to induce persistent immune responses. A holistic understanding of how metabolic heterogeneity governs the immune function of specific T‐cell subsets is key to obtaining field‐level insights into immunometabolism. Therefore, exploring the mechanisms underlying the interplay between T‐cell metabolism and immune functions will pave the way for precise immunotherapy approaches in the future, which will empower us to explore new methods for combating tumours with enhanced efficacy.

## Graphical abstract

Single T‐cell metabolism in tumour immunity

1. Single‐cell RNA sequencing has gained prominence as the preferred tool for clarifying cell subtypes and metabolic heterogeneity.
2. Various metabolic pathways within T cells in the tumour microenvironment are coordinated, leading to diverse T‐cell behaviours.
3. Understanding the mechanisms underlying the interplay between T‐cell metabolism and immune functions will pave the way for precise immunotherapy approaches.

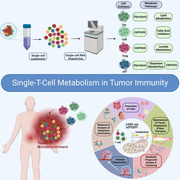

## 1 INTRODUCTION

T cells, essential components of the adaptive immune system, are thought to defend the host from tumour antigen invasion.^1^ In addition, on account of the discrepancies in T‐cell receptor (TCR) antigen recognition and phenotypes, various cytokines or metabolic products in the tumour microenvironment (TME), and the selective effects of tumour cells on immune cells, etc., T cells exhibit great variability in their responses to tumour antigens depending on their various differentiation subtypes and stages. Chronic antigen‐stimulated CD8+ T lymphocytes express high levels of immunosuppressive receptors and are often exhausted, leading to diminished immunological efficacy and decreased cell proliferation capabilities.^2^ CD4+ T‐cell differentiation generates regulatory T cells (Tregs), which are immunosuppressive lymphocytes recruited to the milieu to facilitate tumour cell escape from immune surveillance.^3^, ^4^ Additionally, the differentiation status and specific functions of tumour‐infiltrating T cells (TILs) are related to T‐cell anabolic catabolism and energy metabolism.^1^, ^5^, ^6^, ^7^ T cells undergo metabolic remodelling after activation in response to changes in their surroundings and their own demands, mediating T‐cell immunity through multiple pathways.^8^ For instance, effector T cells (Teffs) rely predominantly on aerobic glycolysis to support their antitumour activity.^9^, ^10^ However, in the TME lacking glucose, Teff glycolysis is reduced, and the amounts of adenosine triphosphate (ATP) and metabolic intermediates produced are insufficient, leading to dysfunction or exhaustion of most CD8+ T cells.^11^ Additionally, the production of T‐cell exhaustion markers in the hypoxic TME is frequently associated with a reduction in oxidative phosphorylation (OXPHOS) in mitochondria.^12^ Fatty acid synthesis promotes the differentiation of CD4+ T cells into helper T cells (Th).^13^ However, Tregs enhance self‐infiltration and help tumour cells escape immune surveillance by activating the transcription factor Foxp3, shifting the energy‐producing metabolic pathway towards fatty acid oxidation (FAO) and OXPHOS to allow adaptation to the lactate‐ and fatty acid‐enriched microenvironment.^14^, ^15^ Moreover, amino acid metabolism in T cells may alter epigenetic modifications, resulting in altered T‐cell function.^8^ T cells can metabolise glutamine to generate S‐2‐hydroxypentanedioic acid(S‐2HG) when the glucose concentration is low, potentially changing the histone and DNA demethylation rates and reprogramming the antitumour immune response machinery in exhausted T cells (Texs).^16^ Furthermore, hypermetabolism in cells in the TME depletes methionine from T cells, which contributes to Teff histone methylation and impairs Teff immunological function.^17^ Substantial variability has been observed among T‐cell subpopulations, and intersubpopulation similarities and differences are often examined at the individual cell and protein expression levels via mass cytometry (CyTOF) and flow cytometry.^18^ CyTOF enables bulk analysis of cellular metabolite abundances, but still lacks of the capability of isolation and definition of individual T‐cell phenotype. Compared to flow cytometry and single‐cell sequencing, CyTOF relies on stringent antibody labelling and single‐cell preparation workflows with lower throughput.^19^ Furthermore, conventional flow cytometry analysis enables the identification of T‐cell subgroups relying on surface markers or molecules. However, it provides limited internal genetic and dynamic real‐time information and fails to support fine‐tuned cell sorting or comprehensive analyses of heterogeneous and complex T‐cell subpopulations in the TME. To push the boundaries of exploring cellular heterogeneity, single‐cell sequencing is gaining popularity as a means for more accurately revealing metabolic variation among various T‐cell subpopulations and serving as an optional technique for in‐depth clinical studies and therapeutic research.

Single‐cell sequencing can be used to obtain DNA and RNA genetic information of individual cells, even when the cellular information exhibits low heterogeneity.^20^, ^21^ In particular, single‐cell RNA sequencing (scRNA‐seq) can be utilised to measure variations in gene expression between samples. Furthermore, the dynamic pathways involved in cell development can be reestablished based on the similarity and heterogeneity of cell expression patterns mined from scRNA‐seq data, and unusual T‐cell subtypes and their differentiation status can be detected.^22^ Specifically, scRNA‐seq‐based findings of different metabolic pathways have revealed that abnormal lipid buildup induces exhaustion in Teffs, causing high‐level lipid metabolism in both Teffs and Texs.^23^ In addition, GADD45β + LAG3 + γδT cells exhibit enhanced glutamine metabolism to maintain survival at the expense of decreasing the expression of cytoprotective molecules.^24^ However, there are controversies regarding exactly how the metabolic status affects immune activity. Notably, scholars have proposed combining metabolic modulators to boost the tumour treatment efficacy based on the metabolic variations among T‐cell subsets identified via scRNA‐seq analysis. For example, JHU083, which antagonises glutamine metabolism by functioning as a glutaminase inhibitor (Gl), drives OXPHOS in Teffs and Th1 cells and promotes the expansion of Teffs and effector memory‐like T cells, thereby inhibiting Treg immunosuppression.^25^

**FIGURE 1:**
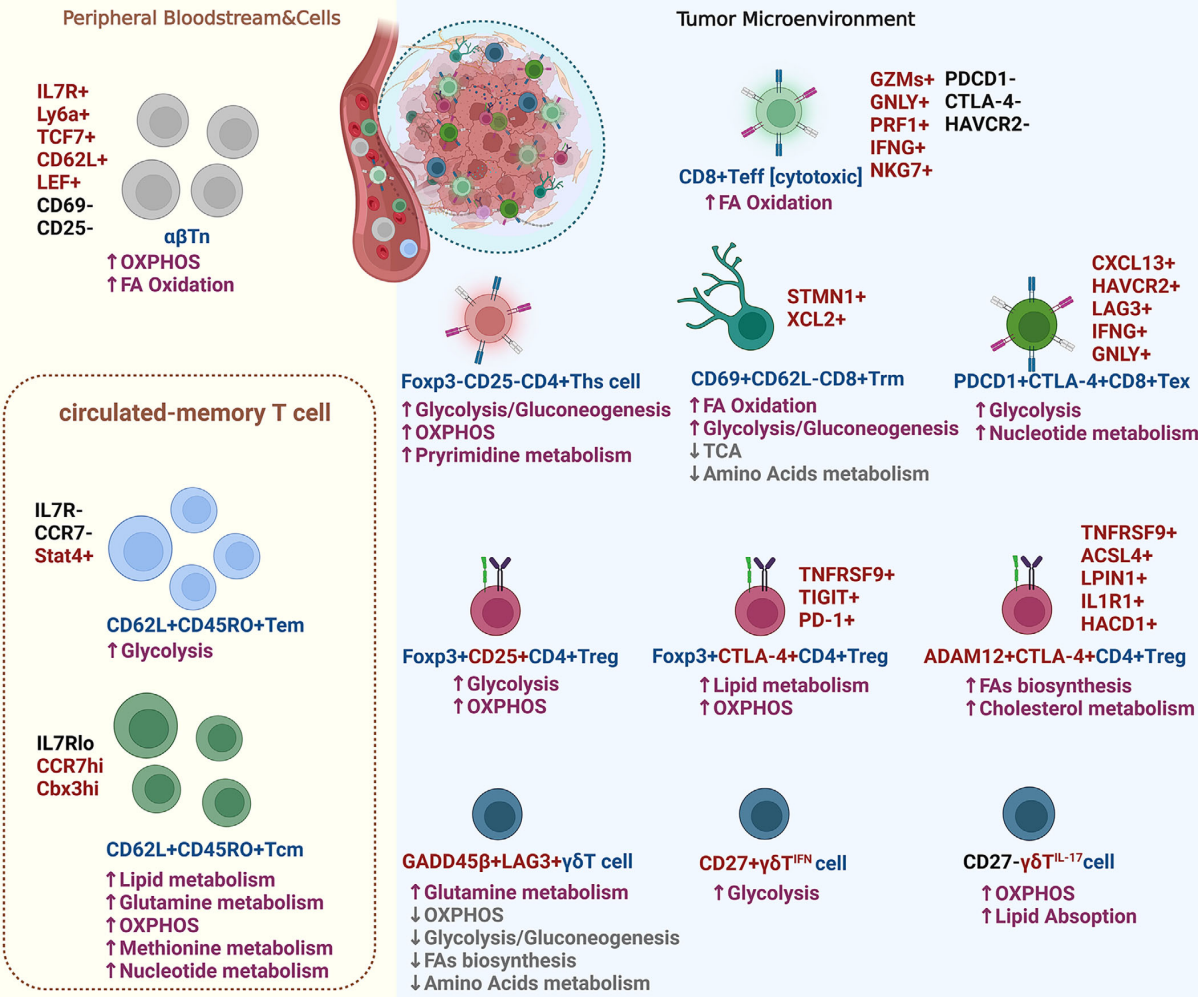
Specific metabolic states of various types of T cells with differentially expressed genes. According to the primitive surface antigen classification, the metabolic features that support differential T‐cell immune activity between and within T‐cell subpopulations are precisely and microscopically characterised based on specifically expressed marker genes, as shown by single‐cell transcriptome sequencing. Red: highly expressed/positive specific markers; black: lowly expressed/negative specific markers; pink: upregulated metabolic pathways; grey: downregulated metabolic pathways. ACSL4: acyl‐CoA synthetase long‐chain family member 4; ADAM12: ADAM metallopeptidase domain 12; Cbx3: chromobox homologue 3; CCR7: chemokine receptor 7; CTLA‐4: cytotoxic T lymphocyte‐associated antigen‐4; CXCL13: chemokine (C‐X‐C motif) ligand 13; FOXP3: forkhead box P3; GADD45β: growth arrest and DNA damage inducible 45 β; GNLY: granulysin; GZMs: granzymes; HACD1: 3‐hydroxyacyl‐CoA dehydratase 1; HAVCR2: hepatitis A virus cellular receptor 2; IL‐1R1: interleukin‐1 receptor 1; IL‐7R: interleukin‐7 receptor; LAG3: lymphocyte activation gene3; LEF: lymphoid enhancer‐binding factor; LPIN1: lipin 1; Ly6a: lymphocyte antigen 6 complex; NKG7: natural killer cell group7; PDCD1: programmed cell death1; PD‐1: programmed death 1; PRF1: perforin 1; Stat4: signal transducer and activator of transcription 4; STMN1: stathmin 1; TCF7: transcription factor 7; TNFRSF9: tumour necrosis factor receptor superfamily 9; XCL2: chemokine (C motif) ligand 2.

At present, systematic reviews using scRNA‐seq to characterise T‐cell metabolism and metabolism‐related immune functions are lacking. In this paper, we review single‐cell sequencing‐based techniques for elucidating the classification of different T‐cell subpopulations and various metabolic reprogramming characteristics of T cells in the peripheral circulation and TME (Figure 1), summarise the factors affecting T‐cell metabolism, and explain how T‐cell metabolism affects antitumour immunity directly or indirectly. Additionally, more reliable T‐cell metabolism studies are providing reasonable explanations for immunotherapy ineffectiveness and revealing therapeutic prospects through which T‐cell metabolism‐regulating pathways exert positive synergistic effects on immunotherapy.

## 2 T‐CELL METABOLIC REPROGRAMMING AND RELATED ANTITUMOUR IMMUNE FUNCTIONS

### 2.1 Circulating T lymphocytes

#### 2.1.1 Metabolism of circulating reactive T cells linked to TILs is crucial for the modulation of antitumour immunity

The peripheral immune system of tumour patients is the basis for the local immune response in the TME.^26^ Communication between peripheral circulating T lymphocytes and TILs following tumour antigen activation is essential for tumour invasion resistance.^27^ After maturation, naïve T lymphocytes (Tns) are dispersed mostly in the peripheral circulation and are activated by tumour antigens as they travel to secondary lymphoid tissues. Activated T cells undergo clonal expansion, proliferation and differentiation and move to nonlymphoid lesion sites facilitated by costimulatory factors and cytokines. Teffs can travel across secondary lymphoid tissues at the early stages of tumourigenesis, but sometimes in nonlymphoid lesions, these cells remain, differentiate locally, and do not reenter the peripheral circulation. While losing the expression of α4β7, T cells acquire the ability to reside and exert effector function in intestinal mucosal tissues.^28^ Those T cells that cease recirculation through nonlymphoid tissues remain stationed at particular sites, serving immune surveillance over the long‐term and retaining memory as well as effector potential. This positioning allows the cells to instantly initiate defensive responses upon direct contact with antigen stimulation.^29^ Interestingly, many studies combining scRNA‐seq and T‐cell receptor sequencing (TCR‐seq) have found that TCR sequences in peripheral blood Tns are identical to those in TILs and that peripheral circulating T cells with this sequence are called circulating tumour‐associated T cells.^30^ These are activated tumour‐specific CD8+ T cells with an effector or effector memory‐like phenotype that are less dysfunctional than clonally expanded CD8+ T cells but show the same time‐dependent degree of exhaustion.^31^, ^32^ The glycosaminoglycan degradation pathway is hyperactivated in circulating tumour‐associated T cells, whereas the purine metabolism pathway is hypoactivated.^30^ In addition, glycosaminoglycan buildup alters the glycoprotein form of cytokines released by T cells, and aberrantly and specifically induces dendritic cells binding to T cells,^33^ which indirectly triggers T‐cell death, facilitating tumour cell immune evasion.^33^, ^34^ In contrast, adenosine (ADO) accumulation appears to preempt amplified immunosuppressive signalling, partially impinging TCR signalling via ADO receptor, thereby dampening T‐cell maturation and diminishing the motility of effector cytokines (interferon‐γ [IFN‐γ], granzyme B, etc.).^35^ Purine metabolism is correlated with ADO production, which is suppressed during one‐to‐one interactions between T cells and tumour cells, efficiently leading to lethality.^36^ According, the reduction of glycosaminoglycan and impediment of ADO production could serve to minimise the accretion of deleterious metabolites that inhibit T‐cell proliferation and activation, which optimises T cells immunological efficacy. Notably, compared with TILs, peripheral blood CD8+ T cells exhibit increases in interleukin (IL)‐2/signal transducer and activator of transcription 5 (Stat5) signalling pathway activation and glycolytic activity after interacting with CD28 costimulatory molecules as well as increases in the electron transport chain and OXPHOS activity levels.^37^, ^38^ Therefore, an increase in the levels of costimulatory molecules is vital for supplying circulating T lymphocytes with energy to boost their antitumour behaviours.

The metabolic rate determines the survival, differentiation and immune function of circulating T cells, which affect tumour prognosis. In the ascites of patients with peritoneal metastases from advanced gastric cancer, researchers have discovered an increased number of nonfunctional proliferating circulating T cells with certain naïve phenotypes (e.g., CCR7, TCF7, LEF1) and high expression of proliferation‐related genes (MK167, STMN1, PCNA).^39^, ^40^ According to a Monocle assay of dynamic developmental pathways in peritoneal circulating T cells, when naïve CD8+ T cells evolve into proliferative circulating T cells, the rates of glycolysis, fatty acid metabolism and nicotinamide adenine denucleotide(NAD) metabolism increase, and their proliferation reaches the maximum rate. In contrast, the cytotoxicity induced by these cells is weakened, and their immunosuppressive functions are enhanced.^41^, ^42^ Directing the rewiring of circulating reactive T‐cell mechanisms and certain metabolic pathways has been speculated to be a promising solution for controlling tumour progression.

#### 2.1.2 The dynamics of circulating memory T‐cell metabolism determine their differentiation status and immune memory formation

Tumour‐specific circulating memory T‐cell (Tm) subsets, including central memory T cells (Tcms), stem cell memory T cells (Tscms) and effector memory T cells (Tems), are the driving forces behind permanent immunological memory formation.^43^, ^44^ Tcms are phenotypically similar to Tn cells in that they rapidly produce IL‐2 in response to TCR activation, leading to the differentiation and generation of many Teffs that migrate to tumour sites, as well as increased phagocytosis and clonal proliferation rates.^45^, ^46^ Tscms are considered precursors of Tcms and are important, in part, as key raw materials for chimeric antigen receptor T cells (CAR‐Ts) with durable immunity,^47^, ^48^ with a transcriptional profile more akin to that of Tns than Tcms, as characterised by higher self‐renewal and cellular plasticity.^43^, ^49^ Without the ability to home to secondary lymphoid tissues, Tems constitute a population that circulates between nonlymphoid tissues and the bloodstream, and in the majority of these cells, effector activities can be instantly induced to enable interception of pathogens.^44^

Tm metabolism is commonly characterised by catabolic processes dependent on FAO and OXPHOS,^50^ but the dependency of distinct subpopulations on energy‐generating pathways varies significantly. According to a chronological analysis of CD8+ T cells performed with early‐stage recurrent hepatocellular carcinoma samples, the initiation of the differentiation phase depends mainly on lipid and lipoprotein metabolism, whereas the effector phase at later stages of development is primarily fueled by fatty acids, implying that early T‐cell differentiation is more closely related to the gene expression phenotype of previously activated CD45RO+ Tms.^39^, ^51^, ^52^ In addition to the shared metabolic patterns among Tm cell subpopulations, the presence of tumour antigens greatly increases nucleotide metabolism during the activation of Tns, which differentiate into Tcms, resulting in Tcms having a much greater self‐renewal ability than Tems. In contrast to Tems, Tcms are more dependent on OXPHOS and FAO, and aromatic amino acid anabolic activity is considerably increased,^53^, ^54^ which confirms previous findings.^55^, ^56^, ^57^ This finding also explains why Tns and Tcms are unable to activate hypoxia inducible factor 1α (HIF‐1α) following exposure to hypoxia; however, HIF‐1α activation in Tems is not restricted by OXPHOS under hypoxic conditions, and these cells obtain energy via HIF‐α‐induced glycolysis.^58^

Multiple metabolic communication pathways disrupt the Tm cell stemness–memory–effector balance. Continuous antigenic stimulation and the inflammatory response in the tumour immunosuppressive microenvironment can lead to metabolic reprogramming of CD8+ Tms cells, which results in their differentiation into end‐stage memory cells; these cells exhibit high cytotoxicity but low proliferative capacity, and Tcms and Tscms undergo apoptosis to promote stem cell differentiation.^59^ Thus, coordinated symbiosis among Tm cell subpopulations is disrupted. The impact of asparagine on T‐cell differentiation depends on its ability to function as a mammalian target of the rapamycin complex (mTORC1) activator, thus dynamically controlling mTORC1 and regulating cellular mitotic spindle formation.^60^, ^61^ However, the timing of asparagine depletion is regulated by *ASNS* overexpression during the predifferentiation stage in CD8+ T cells, which prompts Tcm polarisation and early differentiation, resulting in a delay in Tcm polarisation and driving T‐cell differentiation towards T cells with effector phenotypes.^53^ Furthermore, Tscm synthesis depends on the relationship between metabolic activity and Wnt‐catenin/glycogen synthase kinase‐3/mTORC1 signalling.^62^ Rapamycin, or the Wnt‐β‐catenin signalling pathway activator TWS119, inhibits mTORC1 activity, triggering CD63 expression and activating the transport of fatty acids to intracellular compartments,^63^ endowing Tscms with stable mitochondrial biosynthesis capacity and inducing active lipid oxidation metabolism.^64^ Similarly, impaired amino acid metabolism destabilises the Tm cell amino acid transporter, which on the one hand attenuates mTORC1 signalling, resulting in a slower rate of Tm cell production, and on the other hand, accelerates the acquisition of a effector‐exhaustion phenotype (CXCR3hiCD127lo) in the cells in the peripheral circulation, impeding Teff differentiation into KLRG1‐CD127hi precursor Tm cells (Tpms) and resulting in a decrease in the rate of circulating Tm and a diminished Tm immune response in tumour tissues.^65^ Additionally, 2‐deoxyglucose (2‐DG), which mediates CD8+ T‐cell protein synthesis, influences the memory status.^66^ The T‐cell promoter eIF‐2a phosphorylation level and unfolded protein response are significantly reduced in B16‐OVA tumour‐bearing mice treated with 2‐DG for 36 h, which elevates T‐cell glycolysis and ATP production and release from mitochondria, contributing to CD8+ T‐cell differentiation into Tscms.^67^

In conclusion, the aforementioned studies demonstrated the key role of the T‐cell metabolic pathway in the circulation‐related memory Tcm phenotype, durability and multifunctionality. The few studies conducted to date have been mostly based on animal assays and have thus not studied circulating T‐cell metabolism in the human TME. Murine models have limited ability to faithfully replicate the intricacies of the multifarious antigenic landscape embodied within the human TME. Furthermore, scRNA‐seq enables the acquisition of single T cell with high resolution and authenticity, even identifies emerging subsets in the TME within dynamic shifting of diverse cellular constituents. The high‐dimensional dataset derived from scRNA‐seq bridges the defective information of animal models alone. However, few scRNA‐seq studies have been performed to validate that the adverse environment established by recurrent tumours causes metabolic reprogramming of Tms because of the peripheral circulating environmental pH or nutrient levels.

#### 2.1.3 Flexible shifting between metabolic pathways that depend on resting T cells for required energy

Initially, T cells need high levels of OXPHOS to maintain cell growth homeostasis and differentiation integrity, and following activation, Teffs exhibit reduced mitochondrial respiration rates, whereas glycolysis and glutamine metabolism increase to ensure an efficient energy supply.^68^, ^69^, ^70^ This observation has been validated using scRNA‐seq data together with lifetime imaging of endogenous fluorescence factors.^9^, ^71^ Notably, the rate of OXPHOS in early activated CD8+ T cells potentially fluctuates.^55^ CD8+ T‐cell polyamines also switch from undergoing synthesis to undergoing catabolism during the activation‐to‐differentiation switching phase, which enhances arginine synthesis and urea cycle activity and promotes the survival and memory phenotypic differentiation of cells in the TME.^53^ Furthermore, in an artificially generated high‐fat environment, inactivated T cells efficiently utilise lipids as energy sources, whereas Teffs exhibit increased fatty acid consumption and branched‐chain amino acid synthesis while suppressing proliferation and immunological attack.^72^ The metabolic adaptation of resting and activated T‐cell subsets after exposure to the high‐fat microenvironment show marked differences.

### 2.2 Tumour‐infiltrating T lymphocytes in the immune‐inflamed TME

**FIGURE 2:**
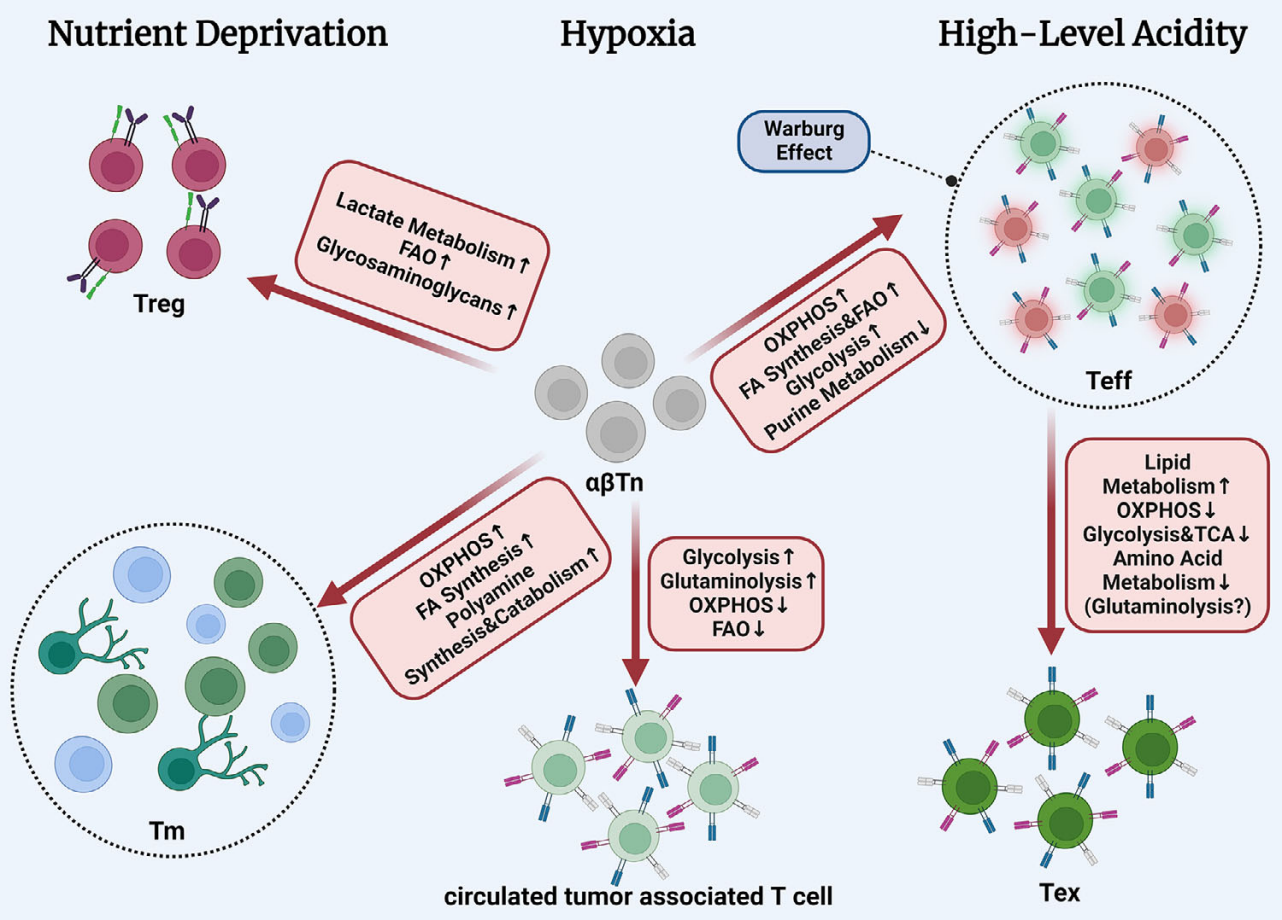
Metabolic changes in the tumour microenvironment (TME) support the differentiation of different T‐cell subpopulations. The metabolic properties of various T‐cell subpopulations show both convergence and substantial variability. In the TME, cancer cells and T lymphocytes compete for metabolic resources. Activated T cells are readily exhausted via hypoxia, excessive acidity, and a lack of nutrients in the hypermetabolic TME, and their metabolic pathways are reprogrammed to respond to these changes in the surrounding environment. Specifically, (1) upon stimulation with tumour‐specific antigens, αβTns undergo metabolic remodelling accompanied by upregulation of oxidative phosphorylation (OXPHOS), fatty acid synthesis and oxidation, which may enhance the infiltration of effector T cells and memory T cells. (2) Circulating tumour‐associated T cells rely on glycolysis and glutaminolysis to sustain their immune activity. (3) Regulatory T cells (Tregs) have the advantage of utilising lactate and fatty acids as energy sources, which facilitates their long‐term immunosuppressive function within the TME. (4) As the overall energetic metabolic activity decreases, effector T cells gradually transition to functionally exhausted T cells.

T cells activated via antigen presentation migrate to and infiltrate the TME to kill tumour antigen‐containing cells by recognising and directly contacting them, releasing cytokines, or activating other immune cells. Based on a universal analysis of scRNA‐seq and exploratory data on downstream genes, it is possible to produce a more precise classification of subpopulations based on conventional surface markers (CD8 and CD4). In addition, the metabolic dynamics of different cell subpopulations and the associated regulatory mechanisms through which these cells adapt to hypoxic, acidic and nutrient‐poor microenvironments during tumour cell competition for resources can be ascertained (Figure 2).

#### 2.2.1 CD8+ T‐cell metabolic diversity

The proliferation of activated T cells necessitates high‐intensity metabolism. Purine nucleotide formation, gluconeogenesis, aerobic glycolysis and FAO are needed for the synthesis of ATP, and amino acid uptake and metabolism have been identified as the major metabolic pathways involved in infiltrating CD8+ T cells based on a Gene Ontology pathway analysis of publicly available multitumour single‐cell transcriptome sequencing data.^73^, ^74^, ^75^ Notably, gluconeogenesis, a metabolic pathway specific to CD8+ T cells, induces tumour cells to acquire abundant glucose, which contributes to cancer cell plasticity, but its effect on CD8+ TILs remains unknown.^76^

##### 2.2.1.1 Complexity of metabolic patterns in CD8+ Teff exhaustion and differentiation trajectories

Increased infiltration of Texs is attributed to aberrant reprogramming of glycolysis and mitochondrial respiration during differentiation.^77^ Teffs that transition to Texs exhibit high expression of cytotoxic and immunosuppressive markers and upregulation of aerobic glycolysis and nucleotide metabolism, which may be closely related to the release of the signalling molecule IFN‐γ from transitional cells and the formation of an inflammatory microenvironment.^73^ However, Texs exhibit low glucose utilisation and mitochondrial respiration rates. Significantly decreased levels of key metabolic enzymes in most energy metabolism pathways, especially aerobic glycolysis and OXPHOS regulators, indicate that T cells are exhausted,^78^ consistent with the results from previous fluorescence‐activated cell sorting studies.^79^ Chronic tumour antigen stimulation interferes with T‐cell adenosine diphosphate (ADP)‐coupled OXPHOS, which directly lowers ATP production and inhibits DNA replication and protein synthesis. Additionally, it induces high‐throughput glucose uptake and redirects glucose towards the lactate production pathway, leading to reductions in the glycolysis and tricarboxylic acid (TCA) cycle rates. As a consequence, the oxygen consumption rate (OCR) and extracellular acidification rate (ECAR) significantly increase in CD8+ T cells, causing these cells to become exhausted, with reduced self‐renewal capacity and, in some cases, a tendency to undergo apoptosis.^80^ Therefore, Teff homeostasis requires a dynamic balance between OXPHOS and glycolysis. Previous studies have indicated that direct interference with mitochondrial ribosomal proteins (CR6‐interacting factor 1, CRIF1) or the CRIF1‐interacting protein Lck,^81^, ^82^ which are associated with tumour pathogenesis,^83^ may provide new insights into the relationship between TILs and mitochondrial protein function via single‐cell multiomics analysis.^84^, ^85^

Lipid metabolic reprogramming plays a key role in Tex differentiation and functional regulation. The exhaustion phenotype of CD8+ Texs exhibit a positive correlation with increased fatty acid synthesis rates, indicating that aberrantly accelerated lipid metabolism is associated with highly dysfunctional Texs.^54^ However, proper initiation of fatty acid metabolism is pivotal for the maintenance of both Teff and Tex immune effects. In an early study of recurrent hepatocellular carcinoma, CD8+CCR6+ T cells, which act as initiators of redifferentiation, differentiated into intermediate cell types in a cytotoxic state with high expression of classical cytotoxic genes (*GZMK*, *GZMH*, *GZMA*) and low expression of exhaustion phenotype‐related genes (*PDCD1*, *CLTA4*, *HAVCR2*). This state was accompanied by elevated FAO, which serves as the main energy source for Teff functions.^39^ Moreover, Fabp5 gene expression specifically increases during CD8+ T‐cell exhaustion, promotes lipid uptake and, more importantly, positively regulates carnitine palmitoyltransferase 1a (CPT1a) activity, thereby activating FAO in T cells. This process confers protection to Texs through the self‐regulation of lipid metabolic homeostasis.^86^ However, the clear activation of fatty acid and cholesterol synthesis pathways results in abnormal lipid accumulation and reprogrammed lipid metabolism, leading to accelerated effector‐to‐exhaustion Tex transitions and the formation of an immunosuppressive microenvironment that contributes to cancer cell growth and proliferation.^23^ Modulating cholesterol metabolism activity through the downregulation of cholesterol synthase activity or modulation of cholesterol uptake and efflux by Teffs can affect the tumour killing efficacy of infiltrating T cells.^87^

Glutamine metabolism is bidirectionally regulated during CD8+ T‐cell differentiation. Tex amino acid metabolism is generally impaired, but glutamine metabolism is more highly active than it is at other stages of the CD8+ TIL transition process.^88^ Notably, the significant upregulation of glutamine metabolism, associated with the activation of the transforming growth factor‐β (TGF‐β) signalling pathway, leads to a reduction in the expression of the tissue‐resident marker CD103 on Texs and induces T‐cell apoptosis and impairment.^89^ Nevertheless, in the colorectal cancer microenvironment, glutamine not only enhances CD8+ T‐cell glycolysis and lactate metabolism but also serves as an essential nutrient for cytokine synthesis.^90^ Thus, the balance of glutamine metabolism is crucial for maintaining T‐cell stability and promoting positive differentiation.

##### 2.2.1.2 Fatty acid and glucose metabolism‐coordinate tissue‐resident memory T‐cell activity

FAO plays a crucial role in supporting the fundamental life activities of tissue‐resident memory T cells (Trms). Trms constitute a Tm population that enters infected tissues during the effector phase of the immune response, ceasing direct involvement in the immune response within the circulatory system but counteracting renewed antigenic attack on local tissue.^29^ A study of the aberrant metabolic microenvironment in the context of multiple myeloma revealed that CD69+STMN1+CD62L–CD8+ Trm cell‐related TCA cycle activity and amino acid metabolism were impaired, whereas glycolysis and FAO were enhanced,^91^ corroborating the pivotal effect of FAO on immune memory mediated by Trms within the TME. More critically, in addition to providing energy support, fatty acid metabolism ensures maintenance of the nicotinamide adenine dinucleotide phosphate(NADPH) and ATP levels in Trms to safeguard them against reactive oxygen species (ROS) peroxidation.^92^ Specifically, the deletion of Fabp4 and Fabp5 in Trms results in reduced uptake of exogenous free fatty acids by CD8+ Trms, substantially diminishing the ability of these cells to survive in the highly metabolic TME.^86^ This outcome aligns with findings from a flow cytometry analysis showing that CD103^hi^ Trms in the TME exhibit a rapid increase in fatty acid uptake, whereas their glucose utilisation rate decreases, contributing to elevations in the OCR/ECAR, spare respiratory capacity (SRC), and maximal respiratory capacity of Trms.^63^ Clearly, Trms within the TME tend to meet their basal metabolic energy requirements predominantly through FAO to prevent apoptosis.

Furthermore, activated gluconeogenesis/glycogenolysis pathways potentially indirectly support the survival and memory functions of Trms. Infiltrating CD8+ Tms within tumour tissues upregulate phosphoenolpyruvate 1 (Pck1) expression, enhance gluconeogenesis or activate the glycogenolysis pathway, activate the pentose phosphate pathway to produce glucose‐6‐phosphate for consumption in NADPH synthesis, and indirectly generate reduced glutathione to defend Trms against oxidative stress.^93^ Regrettably, despite the predominance of evidence showing that infiltrating Tms are Trms, definitive clustering studies following scRNA‐seq remains lacking.

##### 2.2.1.3 Links between immune response duality and metabolism in bystander CD8+ T cells

As a result of interactions between chemokines or cytokines or under the influence of homing‐inducing migration receptors in the peripheral circulation or secondary lymphoid organs, bystander CD8+ T cells, a population characterised by CD39–PD‐1–CD8+ T cells that colonise the TME, passively expand and lack the characteristics associated with chronically stimulated T cells.^94^, ^95^ An exploration of scRNA‐seq and TCR expression data revealed that bystander T cells in the TME, such as those in colorectal and lung cancers, share similarities with Trms and can express costimulatory or coinhibitory molecular signals, but many melanoma‐responsive bystander cells are exhausted.^96^, ^97^ Bystander T cells mainly recognise viral antigenic epitopes not associated with tumours in a local tumour and rapidly secrete cytokines without antigenic stimulation, creating an environment sensitive to tumour‐derived inflammation mediated by Trms. However, bystander T cells cannot efficiently target malignancies due to their inability to detect tumour‐specific antigens and compete for nutrients with strong antitumour Teffs, thus demonstrating their potential to hinder tumour immunity.^96^, ^98^ In addition, studies have highlighted cholesterol synthesis as a metabolic mechanism underlying bystander T‐cell growth, proliferation and IFN‐γ secretion, as detected at single‐cell resolution, which may be related to signalling and immune synapse formation.^99^, ^100^ However, the impact of bystander T cells on Teffs and Trms under conditions of metabolic competition has not been determined. However, further investigations of other subpopulations of bystander T cells are needed to better understand their roles in tumour immunity.

#### 2.2.2 Heterogeneity and convergence of CD4+ T‐cell metabolism data

As key helper cells in tumour cell immunity, CD4+ T cells have dual regulatory functions by either promoting or suppressing CD8+ T‐cell immune responses.^101^ Conventional CD4+ T cells (CD4+ Tconvs) exhibit effector functions by producing various cytokines and directly inducing cytotoxicity.^102^ Tregs, constituting another specific population of CD4+ T cells, are often correlated with poor prognosis and tumour progression.^103^ The aerobic glycolysis and OXPHOS rates are considerably increased when CD4+ Tconvs and Tregs are ready to be activated, and Tregs exhibit increased glucose consumption and mitochondrial activity.^42^, ^104^ Remarkably, CD4+ TILs are more engaged in nucleotide metabolism and exhibit less glutamine metabolism activity than CD8+ T cells are, suggesting that ROS increase IL‐2 release from CD4+ T cells, subsequently causing Th1 cell polarisation and activating cytotoxic CD4+ T cells.^73^, ^104^, ^105^

##### 2.2.2.1 Importance of aerobic metabolism for CD4+ Teff function

Cytotoxic CD4+ T cells rely heavily on aerobic metabolic pathways to perform their core function in cytokine production. After activation of these T cells, the level of the transcription factor helix–loop–helix family member E40 (Bhlhe40) increases. In CD4+ Teffs, the activity of glycolytic enzymes (Pgk1, Eno1, Gapdh, Hk1, Pfkp), which are regulated by Bhlhe40, is decreased, whereas the levels of transcription factors related to mitochondrial metabolism (Cox6a1, Ndufb1‐ps, mt‐Nd3) are reduced, directly impacting the activation of the HIF‐1α signalling pathway under hypoxic TME conditions. These alterations induce an insufficient Teff respiration rate and glucose supply and increase the expression of the cytokines IFN‐γ and colony stimulating factor 2(CSF2) and the cytolytic factor Gzmb.^106^ This study was based mainly on single‐cell transcriptome‐level studies, which indirectly illustrated the metabolic function of T cells. However, further validation through metabolomics is still needed to support these valuable insights. Consistent with previous flow cytometry analysis results, CD4+ Teffs do not exhibit high lactate resistance. In an acidic environment, both glycolysis and mitochondrial respiration in CD4+ Teff cells are profoundly hindered, leading to impaired cytotoxicity.^107^

Glycolytic reprogramming hinders the differentiation of CD4+ T cells into T cells with effector phenotypes. Abnormal upregulation of the immunosuppressive checkpoint programmed death 1 (PD‐1) on CD4+ T cells in the TME results in the inability of glucose‐consuming Teffs to activate the glycolytic effector pathway or to tolerate the low‐glucose immunosuppressive environment, driving them to senescence or exhaustion.^108^, ^109^ In addition, a recent study on the dependence of glycolysis‐related differences in CD4+ T‐cell subsets in the colorectal cancer microenvironment revealed that the transcription factor MondoA or its target protein TXNIP, which drives Treg function, create a low‐glucose environment highly suited for Treg polarisation and mediate Treg secretion of the proinflammatory cytokine IL‐17A. As a result, the formation of many dysfunctional CD4+ T cells is indirectly induced, leading to a continuous imbalance in the immunoregulatory roles of CD4+ T cells.^110^ In comparison to Tregs, proinflammatory CD4+ Th17 cells appear to exhibit high aerobic glycolysis, which is a prerequisite for IL‐17 and IL‐22 production, thereby enhancing self‐renewal and tumour cell proliferation, respectively. CD4+ Th17 cells also play unclear roles in the tumour immune response.^111^ Taken together, these results indicate that stabilising CD4+ Teff glycolytic activity protects Teffs and antagonises the inhibitory effect of Tregs on the TME. In addition, glycolytic metabolism and related signalling regulators may be critical for the interconversion and mutual communication among Tregs and Th17 cells.

##### 2.2.2.2 Differences in glutamine metabolism determine the establishment of specific subpopulations of CD4+ T cells

Th1 and Th17 cell polarisation leads to cells with different degrees of dependence on glutamine metabolism‐related transporters and metabolic enzymes. The action of glutamine in reducing oxidative stress protects Th1 cell mitochondria, facilitating cell differentiation and proliferation.^112^ In the context of amino acid competition in the TME, CTLA4+CD4+ Tregs exhibit accelerated glutamine metabolism, but CD4+ T cells with a low glutamine metabolism rate can take up additional glutamine through the glutamine‐exclusive transporter ASCT2, driving the establishment of a Th1 cell subset and shifting the Th1/Th2 balance in the TME towards Th1 cells to facilitate the recruitment and activation of M1 macrophages and CD8+ Teffs, thereby mediating upregulation of the IFN‐γ response.^88^, ^113^ Notably, Gls increase Th1 effector molecule‐related pathway activation IL‐2/Stat5, the glycolytic metabolism rate, mTORC1/Myc signalling pathway activation and the OXPHOS rate, while Th17 cell proinflammatory activity is decreased.^25^ These findings confirm the metabolic flexibility of TILs in terms of glucose and glutamine interactions.^5^ In TILs, glucose and glutamine regulate each other, endowing these cells with metabolic flexibility. In addition, flow cytometry analysis combined with an assay for transposase‐accessible chromatin using sequencing revealed that Gls and glutamic oxaloacetate transaminase 1 activity directly or indirectly regulate the α‐ketoglutarate/2‐hydroxyglutarate axis (α‐KG/2‐HG axis), regulating chromatin accessibility and epigenetic modifications in Th1 and Th17 cells, thereby finding and tuning the differentiation of Th1 and Th17 cells.^114^, ^115^, ^116^, ^117^ Recent research on metabolic processes has been limited to animal models and in vitro cell systems. To comprehensively elucidate the trends in Th1/Th17 cell differentiation in the context of tumour metabolic competition and the association between metabolic reprogramming and epigenetic marker expression in specific CD4+ T‐cell subtypes, further investigations, particularly single‐cell multiomics analyses, are needed.

Moreover, immunosuppressive signalling in Tregs is controlled by glutamine catabolism. When high levels of glutamine are metabolised in Tregs, an adequate amount of glutamine is produced; glutamine is a potent metabolite that protects Tregs from oxidative stress and regulates the activity of amino acids, such as glutamate, cysteine and serine, associated with Treg infiltration and differentiation.^112^, ^116^, ^118^, ^119^ Inhibition of glutamine transport or antagonism mediated by GI‐induced hydrolysis enables Tregs to survive in a glutamine‐deficient environment, leading to a decrease in the proportion of Foxp3+ CD25+CD4+ Tregs and hindering Treg‐mediated tumourigenesis signalling.^25^ In addition, chromatin immunoprecipitation sequencing revealed that the promotion of glutamine conversion to α‐KG positively regulates Foxp3 methylation by increasing the 2‐HG level, which increases the Th17 cell differentiation rate and disrupts the negative regulation of tumour immune function by Foxp3+CD4+ Tregs.^120^, ^121^ Therefore, glutamine availability is important for Treg‐promoted tumour progression. However, high‐throughput scRNA‐seq data need to be refined to further elucidate the correlation between amino acid metabolism and epigenetic modifications. The results from these analyses will likely support evidence for the bidirectional regulatory role of glutamine and for targeting the balancing point in cellular glutamine metabolism for cancer therapy.

##### 2.2.2.3 Treg‐specific metabolic states to adapt to and enhance the immunosuppressive environment

The coinhibitory effects of Tregs rely on intracellular fatty acid synthesis and metabolism. Compared to CD4+CD25–Foxp3–Tconv cells, CD4+CD25+Foxp3+ Tregs exhibit higher intracellular ATP levels and elevated active glucose metabolism and OXPHOS rates, resulting in increased migration and proliferation potential. However, excessive glycolysis‐induced glucose uptake by Tregs is detrimental to Foxp3 expression, impairing the immunosuppressive function of Tregs.^104^, ^110^, ^122^ In addition, Tregs can drive lipid metabolism to support their own effector cell immune functions. PD‐1 signalling initiates peroxisome proliferator‐activated receptor‐β signalling in conjunction with sterol regulatory element‐binding protein (SREBP) to effectively activate infiltrating Treg fatty acid synthase (FASN), which prompts Tregs to undergo FAO to increase inhibitory signalling stability (as validated by scRNA‐seq).^108^ As an on switch for Treg lipid metabolism and cholesterol synthesis, CD70 interacts with CD27 to induce PD‐1 signalling, help maintain mitochondrial integrity and promote cholesterol and fatty acid accumulation within CD4+Foxp3+CTLA‐4+ Tregs, establishing a shielding system against *IFNG*.^15^ Additionally, an abnormally active lipid metabolism pathway in smoking‐associated specific TNFRSF9^hi^ADAM12+CD4+ Tregs, which serve as the energy source for the transition of cytotoxic T lymphocyte‐associated antigen‐4 (CTLA‐4)+ Tregs into exhausted cells induced by the interaction of ADAM12–ITGB1, was found in a study of T‐cell development trajectories in patients who smoke and have non‐small cell lung cancer, and this high lipid metabolism rate is closely associated with enhanced fatty acid synthesis in terminal ADAM12+CTLA‐4+CD4+ Tregs.^123^ The activation of inhibitory receptors on the surface of Tregs is an essential mechanism underlying immunosuppression, indicating that restricting intracellular fatty acid absorption and consumption by Tregs may reverse immunosuppression in the microenvironment.^124^

Systemic metabolic regulation clearly affects the anti‐inflammatory activity of Tregs because these cells can modulate their own metabolic state by sensing extracellular metabolic behaviours. As major steroid hormones that govern the energy status of the body, glucocorticoids play vital roles in cellular glucose and protein metabolism. According to public database profiles generated from scRNA‐seq data, we found that the glucocorticoid stimulation of Tregs results in overall decreases in cellular TCA activity and the redox system‐glucose metabolism‐linked signalling nuclear factor‐κ‐gene binding inducing kinase/nuclear factor‐κ‐gene binding(NIK/NF‐κB) transduction capacity across cancers.^74^, ^125^ These results suggest that glucocorticoids may positively regulate the translation of HK2, the rate‐limiting enzyme in Treg glycolysis, together with the activation of oxidative metabolism‐related pathways, ensuring a reasonable rate of glycolysis in Tregs and promoting Treg survival and differentiation.^126^ In addition, a scRNA‐seq analysis of T cells after leptin‐engineered lysosomal virus treatment revealed that leptin, an anabolic enhancer and potent metabolic regulator, contributes to the maintenance of T‐cell mitochondrial stability and inhibits the immunosuppressive function of Tregs by reprogramming lipid metabolism.^127^, ^128^ In general, metabolic signalling associated with biological rhythms may be a promising approach for attenuating TME cell metabolism, which may lead to significant advancements in cancer treatment and immune modulation by regulating Treg energy metabolism.

Lactate acts as a Treg‐specific carbon metabolism hub, endowing Tregs with metabolic flexibility. The unhindered formation of the immunosuppressive microenvironment is attributed in part to the paradoxical action of lactate in inhibiting CD8+ Teff migration while supporting CD4+ Treg survival.^129^ Considering the scRNA‐seq data, we reproduced and examined a heterogeneous group of Foxp3+ NKTregs that express high levels of CD4, CD25R, Foxp3, programmed cell death1 (PDCD1), CTLA‐4, inducible co‐stimulator(ICOS) and T cell immunoreceptor with Ig and ITIM domains(TIGIT), among others, and acquired a phenotype very similar to that of inhibitory CD4+ Tregs in depth; these cells proactively exploit favourable conditions, efficiently convert lactate to pyruvate, and subsequently enter the mitochondrial TCA cycle.^130^ Furthermore, Tregs actively absorb lactate by triggering monocarboxylate transporter 1 (MCT1) to indirectly limit PD‐1 expression on Teffs.^131^ An increase in MCT1 activity positively regulates the ability of Foxp3–NKTregs to utilise extracellular lactate, and overexpression of lactate dehydrogenase B leads to an increases in the intracellular lactate conversion rate, cellular oxygen consumption and mitochondrial activity, thereby reinitiating the differentiation of Foxp3+ NKTregs.^107^, ^130^, ^132^ In addition, phosphoenolpyruvate can be synthesised via the carbon of lactate and can be produced as an intermediate of glucose and glycogen metabolism in Tregs. First, lactate can alleviate Treg sensitivity to high glycolysis, and second, it supplies the fuel necessary for Tregs to enter the TCA cycle, thereby promoting their proliferation and tumour growth‐promoting capacity.^132^ These findings clearly corroborated the finding that lactate is an alternative fuel source for mitochondrial activity but did not confirm that Treg survival is entirely dependent on lactate.^133^ A recent SMART‐seq‐based study revealed a positive correlation between PD‐1 expression and cellular lactate uptake, suggesting that the effectiveness of anti‐PD‐1 therapy may be influenced by the level of lactate metabolism in Tregs.^131^ In summary, the relationship between Treg lactate metabolism and PD‐1 expression may have implications for immune checkpoint blockade (ICB) strategies in cancer treatment.

#### 2.2.3 The rapid response of innate‐like T cells accompanies metabolic reprogramming

Innate‐like T cells (ILTCs) are direct effector cells that bridge innate and adaptive immunity and are highly sensitive to changes in the surrounding environment, and these cells rapidly secrete inflammatory molecules and tissue‐protective factors.^134^ Distinctions between the TME and a normal environment are rapidly sensed by cytokine receptors on the surface of ILTCs, triggering metabolic reprogramming to maximise the adaptation of these cells to environmental changes and thus fulfilling their roles in tumour immune surveillance, thereby connecting the intrinsic and adaptive immune responses.^135^

The developmental and functional status of ILTCs in the thymus is closely associated with metabolic reprogramming. Defects in the oncogene *PTEN* act as risk factors for tumourigenesis due to loss of the inhibitory effects of these defects on phosphatidylinositol 3‐kinase (PI3K)/protein kinase B (Akt)/mTOR signalling.^135^ Similarly, *PTEN* deficiency in type 17 ILTCs in the thymus of mice is associated with decreased OXPHOS, glycolysis and nucleotide metabolism rates and an increased rate of steroid synthesis in CCR6+CD27+TCRβ+iNKT17/MALT17 cells. These changes lead to decreases in the mitochondrial activity and viability of iNKT17 and MALT17 cells, implying that metabolic processes during the proliferative phase of ILTCs are uncontrolled in *PTEN*‐mutant tumours.^136^ This study also revealed that *PTEN*‐deficient thymocytes exhibit downregulated mitochondrial activity mediated through upregulation of the P13K–mTORC coordinating mechanism or the IL‐23R/Stat3 signalling pathway. These pathways may contribute to the maturation and accumulation of type 17 ILTCs in the thymus and the development of quiescence, potentially allowing tumour cells to evade immune surveillance.^136^, ^137^

The immune response of γδT cells is intricately linked to their coordinated carbon and nitrogen metabolism pathways. This cell subset reaches their target‐of‐action threshold by adapting to the constant stimulation of phosphorylated antigens (PAgs) produced by tumour cells, resulting in a low immune response and exhaustion of the cells as they infiltrate the TME.^138^ For example, the Foxg45β+LAG3+γδ T cells of patients with hepatocellular carcinoma fails to produce cytotoxic molecules, such as IFN and granzyme B, and their glycolysis and OXPHOS rates as were as their rates of fatty acid and multiple amino acid synthesis are markedly decreased. Surprisingly, the glutamine transporter SLC1A5 appears to be active, positively shifting metabolic substrates towards glutamine pathways, additionally suggesting a near loss of effector function.^24^ γδT cells in the TME tend to adjust and rely on glutamine metabolism to meet basic survival needs via nitrogen metabolism and to maintain intracellular nucleic acid and amino acid synthesis.^139^ In addition, recent studies have demonstrated that the dual role of γδT cells is attributable to the production of different effectors^140^ and that subpopulations of γδT cells with different cytokine production programs have different metabolic pathway requirements, leading to heterogeneous metabolic remodelling of γδT cells in the TME. Moreover, analyses integrations scRNA‐seq data and mitochondrial membrane potential data have revealed that tumour‐infiltrating γδT cells (CD27+γδT^IFN^) are mostly dependent on glycolysis and express high levels of Myc, whereas γδT cells that predominantly secrete IL‐17 (CD27‐γδT^IL‐17^) are involved in mitochondrial oxidative metabolism and show the capacity for the specific uptake of fatty acids and cholesterol.^141^ Inactivation of the glucose transporter Glut1 results in reduced IFN‐γ levels without affecting the IL‐17 secretion rates, which supports these findings.^140^, ^142^

**FIGURE 3:**
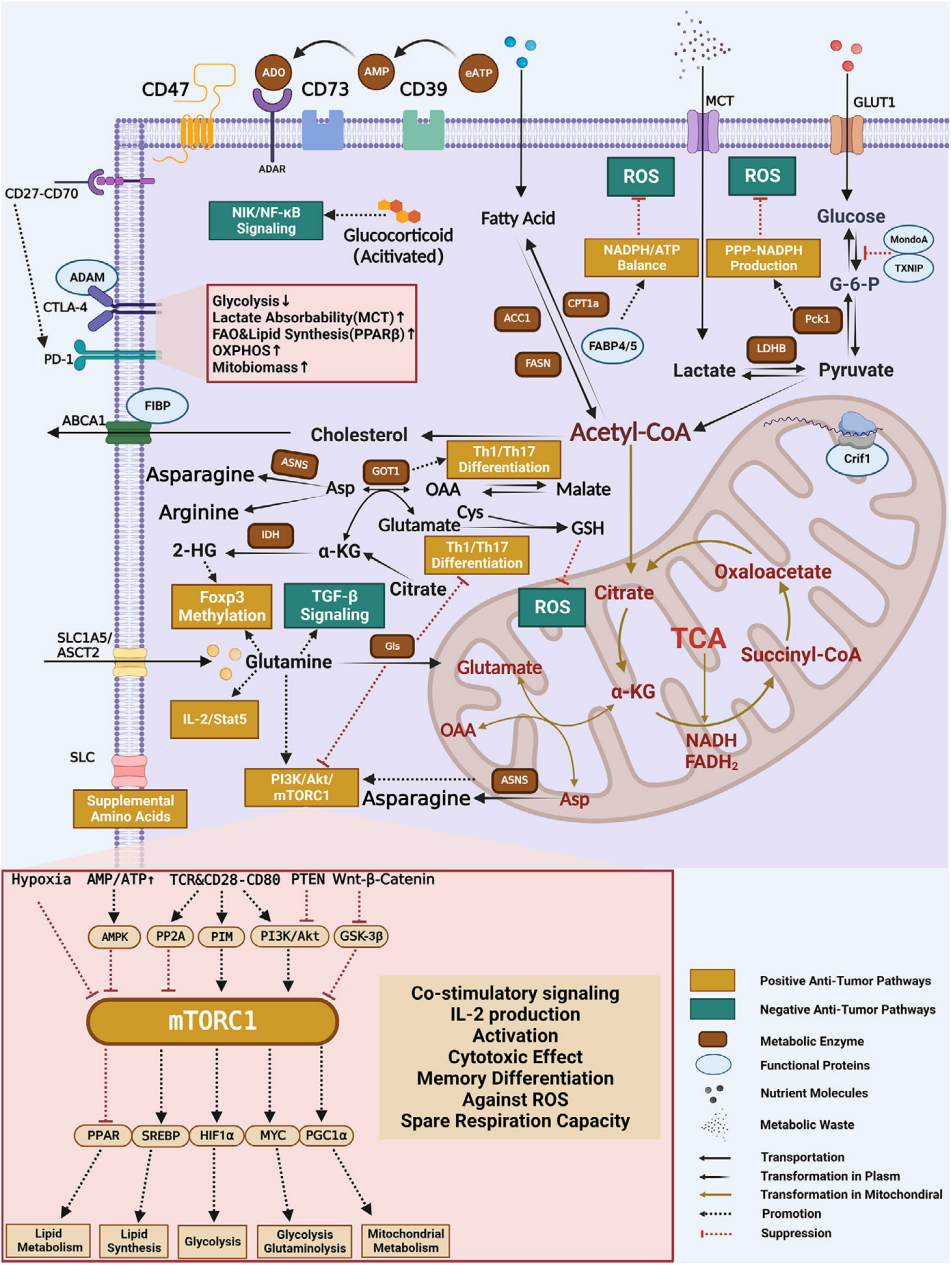
Targets, signalling pathways and mechanisms potentially regulating T‐cell metabolism. Glucose, lipid and amino acid metabolism are intricately linked in the T‐cell metabolic environment. Targeting key metabolic enzymes, transporter proteins and the protein translation machinery can directly or indirectly influence T‐cell immune function related to metabolic substrates and intermediates. Mammalian target of rapamycin complex 1 (mTORC1), an important mTOR complex, plays a crucial role in the intricate metabolic network. The panel below the figure highlights its intermediary role. Specifically, mTORC1 can be activated by different kinases and environmental signals and subsequently modulate metabolism‐related transcription factor expression, thereby altering metabolic processes related to immune function, including cell growth, proliferation and differentiation, and cytokine production. Cell surface proteins (CD47, CD39 and CD73), immune checkpoints (programmed death 1[PD‐1] and cytotoxic T lymphocyte‐associated antigen‐4[CTLA‐4]), and costimulatory ligands and receptors (CD27–CD70) all play important roles in T‐cell metabolism control and can even directly modify T‐cell‐dependent metabolic pathways. Furthermore, T‐cell metabolic pathways are intimately connected to the activation of associated positive/negative antitumour pathways and balance T‐cell immune activity at multiple levels. ADAR: adenosine deaminase receptor; ADP: adenosine diphosphate; Akt: protein kinase B; AMP: adenosine monophosphate; AMPK: adenosine 5′‐monophosphate (AMP)‐activated protein kinase; ASNS: asparagine synthetase; A1FIBP: Fibroblast Growth Factor Intracellular‐Binding Protein; GLUT1: glucose transporter 1; GOT1: glutamic oxaloacetic transaminase 1; GSK‐3β: glycogen synthase kinase‐3β; HIF‐1α: hypoxia inducible factor 1α; IL‐2: interleukin‐2; LDHB: lactate dehydrogenase B; OAA: oxalacetic acid; PGC‐1α: peroxisome proliferators‐activated receptor‐γ coactivator‐1α; PI3K: phosphatidylinositol 3‐kinase; PPAR‐β: peroxisome proliferators‐activated receptor‐β; ROS: reactive oxygen species; Stat5: signal transducer and activator of transcription 5; TCR: T‐cell receptor; TGF‐β: transforming growth factor‐β; TXNIP: Thioredoxin‐Interacting Protein.

In summary, all the dynamic changes in the effector states of different TILs, the interconversion of cell subpopulations during development and differentiation, and the coordinated mechanisms underlying anticancer or cancer‐promoting functions rely on similar or different metabolic pathways to some extent (Figure 3). Understanding the metabolic processes that govern T‐cell subsets may provide crucial insights into the mechanisms underlying human tumour progression and immunotherapy tolerance at the cellular metabolism level.

## 3 THERAPEUTIC ADVANCES IN TARGETING T‐CELL METABOLIC PROCESSES

Based on the differences in metabolic pathways enriched by the transcriptome expression of different T‐cell subpopulations, a growing number of studies have successively proposed that metabolic pathways involved in the effector pathways of T‐cell activation and development, differentiation and cytokine production may be mechanistic targets for enhancing T‐cell antitumour immunity (Table 1).

**TABLE 1:** Therapeutic methods targeting T‐cell metabolism.

	Targets	Measurements	Effects of T cells	Experimental models	Ref.
Regulating metabolitic pathways					
Aerobic glycolysis	eIF‐2a phosphation ↓ UPR ↓	2‐DG	Proteins translation rate↑ Differentiate stem‐like memory T cells Maintain stemness and effector persistence of CAR‐Ts	Mices	^64^, ^143^, ^144^
Glycolysis, FAO	mTORC1 ↓	Rapamycin	Induce CD95+CD58+CD62Lhi+Tscm Long‐lived proliferation and improved self‐renewal	Mices	^61^
Glycolysis, FAO	mTORC1 ↓ GSK‐3β ↑	TWS119 (Wnt activator)	Induce CD95+CD58+CD62Lhi+Tscm CD63 and IFN‐response gene expression upregulated Positively regulate T‐cell immune response	Mices	^61^
Glycolysis, FAO	AMPK ↑	Metformin	Positively induce Tcm and Tscm differentiation Essential for long‐term survival and effector persistence Constrain Foxp3 and CTLA‐4 expression of Tregs	Mices	^145^, ^146^, ^147^
Glucocorticoids activation	HSD11B1 ↓	HSD11B1 inhibitors	Enhance IFN‐γ secretion from CD8+ T cells treated by ICIs Locally decrease reactive glucocorticoids	Cutaneous melanoma cell lines	^148^
FAs biosynthesis	ACC1 ↓	TOFA (ACC inhibitor)	Form CD4+ Tpm (CD62Lhi IL‐7Rαhi CD137lo CCR7hi CD137lo) with strong respiratory capacity Enforce memory Th1 differentiation	Mices	^149^
Glutamine metabolism	Gls ↓	TGF‐β neutralisation	Instabilise Treg phenotypes and complete Treg‐Tex transition (GZMB, PRF1, IFNG, PDCD1, LAG3 upregulated and Foxp3, CTLA‐4 downregulated) CD103 expression upregulated in CD8+ Tex for residence	HNSC samples (human)	^86^
Glutamine metabolism	Gls ↓	JHU083‐EVax (Gl inhibition) DRP‐104 (broad glutamine antagonist)	Polarise antitumour Th1 effectors and negatively alter pro‐tumour Th17 differentiation Increase Th1 expansion by sensitising Th1 to mTORC1 signalling activated by IL‐2 Maintain long‐term immune memory Prevent immunity and surveillance from immunosuppressive cells	Mices and EGFR MT LUSC cell lines	^24^
Glycolysis, amino acid metabolism, OXPHOS	mTORC1 ↓	AZD1208 (PIM kinase inhibitor)	Maintain CD8+ Teff potent immune responses and persistence Tune the phosphorylation of various proteins or substrates (Foxo, Akt, S6) Positively differentiate Tn into CD62Lhi+Tcm and sustain long‐term memory responses	MM samples (human)	^88^
Glutathione synthesis		N‐AC	Anti‐ROS attack and maintain ADP‐coupled OXPHOS Inhibit T cells exhaustion Restore T cells self‐renewal and stemness	Cell lines	^77^
Tryptophan metabolism		Metformin	Control the metabolitic competition of tryptophan to restore the level of tryptophan of CD8+ T cells Positively alter CD8+ T cells fate	Mices	^150^
Methionine metabolism	H3K79me2 ↑ pStat5 ↑	Methionine	Sensitise T cells to mention the deprivation of methionine Protect epigenetic signatures to stablise immune functions Lower the level of PD‐1 in CD4+ T cells	Mices and tumour samples (human)	^18^, ^151^
Glutamine metabolism	Gls ↓	CB‐839 (Gl inhibitor)	Increase the cytotoxic factors released by CD8+ T cells Weaken Teff differentiation and cytotoxicity under PD‐1 blockade^a^	KRAS MT LUAD mices with Keap1 and STK11/Lkb1 co‐MT	^152^
Arginine metabolism	Arg1 ↑	OAT‐1746 (arginase inhibitor)	Restore antitumour potential of CD8+ Teff Constrain Foxp3 expression of Tregs Increase immunotherapeutic benefit combined with PD‐1 mAbs and STING agonist	Mices	^153^
Directly recoding gene expression					
Glutamine absorption	ASCT2 ↑	ASCT2 activation	Positively alter Th1 production and effector function Th1s secret IFN‐γ and IL‐2 to recruit toxic T cells and antitumour macrophages Decrease CD25+Foxp3+ CD4+ Treg production and pro‐tumour pathways activation in TME	LUAD cell lines (human)	^85^
Asparagine metabolism	ASNS expression ↑	ASNaes	Regulate Tm differentiation direction by determining the time asparagine use up Balance stemness and effective ability of CD8+ T cells	Mices	^39^
Amino acids absorption	SLC7A1, SLC38A2 ↑	Pofut1 depletion	CD8+ Teff proliferation and lasting activity of metabolism Teff–Tm transition Enhanced both Teff and Tm toxicity and antitumour immunity Stimulate mTORC1 signalling	Mices	^62^
Mitochondrial metabolism	Leptin ↑	Leptin‐expressing vaccinia virus	Increase KLRG1hiCD127+Tpm and TCF7+CD8+Tcm differentiation Decrease Treg production Support metabolitic requires of new TILs	Mices	^23^
Lactate metabolism	MCT1 ↓	MCT1 knockout	Weaken acidity of Tregs for multi‐metabolism destruction Blockade lactate‐induced glyconegenesis to constrain Tregs energy needs and proliferation	Mices	^125^
Cholesterol metabolism	ABCA1, LDLR, SPEBF2, cholesterol enzymes expression ↓	Fibp knockout	Promote CD8+ T cells antitumour efficiency and effects Partially sustain cholesterol synthesis for Teff activation	Mices	^84^
Aerobic glycolysis	Eno1, GAPDH, HIF‐1α ↑ Cox6a1, mt‐Nd3, Ndufb1‐ps ↑	Bhlhe40 activation	Product energy source for Teff and Tm differentiation Recode metabolism due to hypoxia‐related signalling Improve therapeutic effects of ICTs	Mices	^42^
Glycolysis	IL‐17A ↑	MondoA–TXNIP axis depletion	Overdue glycolysis activity to induce Tregs exhaustion (Foxp3 downregulated) Improve IL‐17A secreted by Th17‐like Treg to exhaust CD8+ Teff	Mices	^107^
Glycolysis	CD47 ↓	CD47 depletion	Improve granzyme B secreted by CD8+ T cells Prevent tumour from being independent of immunosurveillance A potential target of PD‐1 mAbs for better immune benefits	Mices	^154^
Glycolysis	PP2A complex assembly	ZEP knockout	Boost CD8+ T cells antitumour efficiency and effects Promote cytokine and IFN‐γ release Restrian mTORC1‐mediated metabolitic reprogramming	Mices and CRC samples (human)	^155^
Target ACT					
Mitochondrial metabolism	PD‐1 ↓	PDCD1 KO CAR‐T	Improve spare respiratory capacity of CAR‐Ts Enhance ATP production and metabolitic flexibility Activation and immune response with higher quality Enhance mitochondrial fitness of 4‐1BB CAR‐Ts	Tumour cell lines (human)	^156^
Mitochondrial metabolism, amino acid metabolism		PRODH2 KI CAR‐T	Enhance mitochondrial fitness of CAR‐Ts Enhance tumour cytolysis and killing capacity Maintain memory‐like signatures and effectors potential	Mices	^157^
FAO	CPT1a, PPAR‐γ, CD137, Bcl‐2 expression ↑	FABP5high CD137 CAR‐T	Long persistence of TILs Promote proliferation and anti‐apoptosis of T cells Balance lipid metabolism to prevent ROS attack and stress increase	HCC samples (human)	^83^
Mitochondrial metabolism		ATPIF1‐overexpressing CAR‐T	Increase Tcm and Tscm differentiation Increase IFN‐γ and granzyme B released by CAR‐Ts Reduce IL‐2 expression to maintain the effector phenotype Enhance CAR‐T proliferation	Mices and tumour cell lines (human)	^158^
Glycolysis	TGF‐β/Smad signalling	TGF‐βR2 KO CAR‐T	Contain Foxp3 transcription and expression in CAR‐Ts Inhibit CAR‐T exhaustion Eliminate tumours with higher efficacy and efficiency Weaken the stress of glucose competition in TME	Mices and tumour cell lines (human)	^159^, ^160^, ^161^
Glycogen and FAs storage		Glut3‐overexpressing ACT	Accumulate energy for engineering T cells in TME Uptake glucose without uncontrolled activation Promote GSH/GSSG ratio to quench ROS	Mices	^91^
Glycolysis, PPP	NIK ↑	NIK activation Ectopic NIK‐expressing ACT	Stablise HK2 translation Maintain G6PD–NADPH redox system to control intracellular levels of ROS Improve IFN‐γ production capacity of CD8+Tex	Mices	^121^
Lactate metabolism	LDHB↑	LDHB‐overexpressing ACT	Promote lactate–pyruvate transition to counteract the uptakes of lactate and protons Prevent accumulation of lactate in T cells and acidity from impairing immune efficacy High NADH and OXPHOS No significance for Treg differentiation	HCT116 spheroid (tumour cell lines co‐cultured)	^104^
Target ICBs					
Glutamine metabolism	LAG3 ↓	LAG3 depletion	Restore CD4+ and CD8+ T cells immune efficacy and cytotoxicity Positively alter γδT cells killing effects Prevent glutamine from exhaustion Possibly and largely enhance therapeutic effects of CAR‐γδT cell	HCC samples (human)	^131^
FAO, lipid absorption	PD‐1 ↓	PD‐1 depletion	Treg‐related markers downregulated Disturb Tregs proliferation and immunosuppressive effects	Mices	^16^
FAs biosynthesis	PD‐1 ↓	PD‐1 depletion	Protect Teff from CTLA‐4A+Tregs‐induced cell–cell adhesion	NSCLC cell lines (smoking humans)	^120^
FAs biosynthesis, cholesterol metabolism	CD70–CD27 ↓	CD70 mAbs	Break the lipid signalling to disrupt fitness and flexibility of Treg in TME Restore immune surveillance	NPC samples (human)	^16^
ATP–ADO metabolism	CD73–CD39 ↓	CD73 mAbs	Prevent cytotoxicity of Teff from the accumulation of ADO Maintain extracellular ATP to keep antitumour responses	Mices and tumour samples (human)	^33^, ^162^, ^163^, ^164^
ATP–ADO metabolism	CD73–CD39 ↓	CD39 mAbs	Prevent cytotoxicity of Teff from the accumulation of ADO Maintain extracellular ATP to keep antitumour responses	Mices and tumour samples (human)	^33^, ^162^, ^163^, ^164^

Abbreviations: ACC1, acetyl coenzyme A carboxylase 1; ADO, adenosine; ADP, adenosine diphosphate; Akt, protein kinase B; AMPK, adenosine 5′‐monophosphate (AMP)‐activated protein kinase; ATP, adenosine triphosphate; CAR‐T, chimeric antigen receptor T cells; CTLA‐4, cytotoxic T lymphocyte‐associated antigen‐4; FAO, fatty acid oxidation; FA, fatty acid; Gl inhibitor, glutaminase; GSK‐3β, glycogen synthase kinase‐3β; ICB, immune checkpoint blockade; ICI, immune checkpoint inhibitor; IFN; interferon; IL, interleukin; mTORC1, mammalian target of the rapamycin complex 1; OXPHOS, oxidative phosphorylation; PD‐1, programmed death 1; PPAR‐γ, peroxisome proliferators‐activated receptor‐γ; PPP, pentose phosphate pathway; PP2A, protein phosphatase 2A; PRODH2, proline dehydrogenase 2; GSH, glutathione; GSSG, oxidized glutathione; ROS, reactive oxygen species; Tcm, central memory T cell; Teff, effector T cell; Tem, effector memory T cell; Tex, exhausted T cell; TGF‐β, transforming growth factor‐β; Th, helper T cells; TIL, tumour‐infiltrating T cell; Tm, memory T‐cell; TME, tumour microenvironment; Tpm, precursor Tm cell; Tscm, stem cell memory T cell; 2‐DG, 2‐deoxyglucose; NSCLC, non‐small cell lung carcinoma; HCC, hepatocellular carcinoma; LUAD, lung adenocarcinoma; HNSC, head an neck squamous cell carcinoma; CRC, colorectal cancer; MM, multiple myeloma; ACT, adoptive cell trasfer therapy; ICT, immune checkpoint‐targeted therapy; NPC, nasopharyngeal carcinoma; UPR, unfolded protein response.

^a^ Negative effects of T cells.

### 3.1 Targeted regulation of T‐cell metabolism enhances T‐cell antitumour immunity

The PI3K/Akt/mTOR signalling pathway is involved in the complex metabolic network of T cells, mediating TCR rearrangement and directly regulating the uptake, transport and catabolism of various nutrients.^165^ mTOR signalling integrates signals derived from energy and nutrients, and its activity is associated with serine/threonine protein kinase activity, upstream activation of Akt could directly phosphorylate and induce the nuclear export of FoxO transcription factors, suppressing the translation of glycolytic inhibitory genes.^166^ Although mTOR signalling inhibition is broadly considered immunosuppressive, it can contribute to enhanced immune responses in some environments.^167^ The attenuation of mTORC signalling may enhance costimulatory signalling, thereby endowing T cells with a higher SCR and increasing T‐cell memory persistence,^64^, ^145^, ^168^ and it may also lead to a disproportionate Teff contraction/expansion ratio.^146^ The mTOR complex, particularly mTORC1, is the pivotal intermediate for orchestrating anabolism and catabolism, on the one hand, it could mediate HIF‐1α, c‐Myc, etc. to facilitate glycolysis and amino acid metabolism. On the other hand, it could activate downstream SREBPs and participate in the expression of fatty acid synthesis enzymes (FASN, ACC, etc.), regulating lipid metabolism.^147^, ^169^ PIM kinase, a member of the serine/threonine kinase family, controls the phosphorylation of various proteins involved in cellular metabolism and survival and is closely associated with mTORC1 signalling activity to influence CD8+ T‐cell memory state formation.^170^ The pan inhibitor of the PIM kinase AZD1208 inhibits mTOR signalling activity, preventing ROS interference with CD8+ Teff mitochondrial metabolism and restoring amino acid transport, thereby increasing CD8+ T‐cell toxicity and promoting Tm proliferation and persistence.^91^ In addition, metformin phosphorylates AMPK to downregulate mTORC1 and induce the glycolysis‐dependent conversion of CD8+ T cells to FAO, contributing to enhanced acquisition of a memory phenotype and prolonged cell survival.^149^, ^155^ In contrast, metformin positively reprograms the glycolytic pathway in Tregs and weakly inhibits Foxp3 and CTLA‐4 phenotype‐related signalling.^171^ Therefore, data on transcriptional and epigenetic levels are needed to determine the mechanisms underlying the regulatory effects of metformin on the AMPK/mTORC1 axis and corresponding metabolic reprogramming. Undeniably, metformin, which has been evaluated in preclinical studies, may be a metabolically activating small‐molecule drug that increases tumour immunity.^172^, ^173^ In contrast, TCR‐induced zinc finger protein 91 (ZEP91) translocation promotes assembly of the serine/threonine protein phosphatase 2A complex, which regulates mTOR signalling. The knockdown of *ZEP91* in TILs allows cellular effector functions to be fully realised due to mTORC1 activation during hyperglycolysis.^148^

Lipid metabolism is needed for T‐cell activation and long‐term survival, and the interconversion between lipid metabolism and glycolysis characterises the T‐cell effector state. Among the factors involved in this transition, reprogrammed fatty acid metabolism controls memory phenotype development and the functional expression of genes. Inhibition of the fatty acid synthesis pathway is mediated by naïve CD4+ Tn acetyl coenzyme A carboxylase 1, which turns these cells into CCR7^hi^CD137^lo^CD4+ Tpms. These cells can potentially differentiate into Th1 cells with competent memory function and high respiratory capacity, which are central to the TME.^174^ Recent studies have demonstrated that maintaining a competent memory state by satisfying CD103+CD69+CD8+ Trm fatty acid metabolism may prevent the exhaustion of converted EOMES+CD8+ T cells, thus indirectly attenuating acquired epidermal growth factor receptor(EGFR)‐tyrosine kinase inhibitor resistance and the ICB treatment response.^151^ In addition, as previously mentioned, cholesterol metabolism has been implicated in adverse influences on CD8+ Teff‐related antitumour immunity. CD8+ T cells with inhibited or knocked down FIBP exhibit limited cholesterol accumulation due to blocked cholesterol synthase gene expression and increased protein expression of the cholesterol efflux pump protein ATP‐binding cassette transporter ABCA1.^87^ Admittedly, statins used to reduce the cholesterol levels in preactivated T cells prevent Tns from receiving antigenic stimulation and thus inhibit their proliferation.^87^, ^175^ Similarly, activated glucocorticoids not only regulate Treg glucose metabolism but also inhibit antigen‐specific T‐cell responses.^150^ 11‐β‐Hydroxysteroid dehydrogenase‐1 (HSD11B1) converts intracellular inert glucocorticoids into active glucocorticoids, not systemic glucocorticoids. Pharmacological inhibition of HSD11B1 protects against immune surveillance and the tumour‐killing capability of TILs and increases IFN‐γ signalling to induce immune checkpoint inhibitor (ICI) responses but possibly exacerbate the risk of immune‐related adverse events (irAEs).^176^

Exogenous supplementation of nutrients consumed during TIL competition exerts a positive effect by restoring initial immune effector function. SAM, for example, is a substrate of histone methylases in CD8+ T cells, establishing a connection between methionine metabolism and epistatic modification.^153^ Supplementation with methionine inhibits the ongoing inhibitory effects of T cells on methionine competition and restores normal H3K79 methylation to the level of S‐adenosylmethionine(SAM) at the intracellular level.^17^ This restoration of cell competition for methionine inhibits the activation of CD4+ T cells induced by immunosuppressive interacting pairs to some extent.^177^ Although changes in T‐cell epigenetic and metabolic patterns jointly determine T‐cell differentiation, such as the transition into T cells with an effector, memory‐maintaining or exhausted phenotype, whether increase in epigenetic remodelling or metabolic processes can completely reverse T‐cell dysfunction remains unclear.^152^ Through analyses of scRNA‐seq data, the restoration of tryptophan availability to CD8+ T cells has been shown to be achieved via the control of metabolic tryptophan competition by metformin in the TME, thereby changing the CD8+ Teff fate.^162^ N‐acetylcysteine restores the self‐renewal‐dependent stemness of depleted T cells by increasing the intracellular calcium load and redox capacity and enhancing aerobic glycolytic metabolism to produce ATP.^80^ In addition, the antitumour activity of CD8+ T cells is dependent on the intracellular L‐arginine concentration. The uptake of available L‐arginine accelerates CD8+ Teff OXPHOS and TCA cycle progression, which protects cells from the malignant consequences of the tumour Warburg effect by inhibiting glycolytic metabolism. When the L‐arginine concentration is elevated to a specific level, Teff differentiation into Tcms is accelerated to optimise TME survival.^154^ Notably, the arginase inhibitor OAT‐1746 likely induces a better therapeutic response compared with a double‐drug regimen consisting of an ICI and the STING agonist DMXAA to activate the intrinsic immune response, but this regimen fails to overcome the inadequate immune effects.^178^ In conclusion, more precise amino acid‐related immune metabolic process probes must be developed to provide guidance for clinical trials of innovative antitumour drugs.

The dual effect of adequate glutamine suggests that glutamine metabolism‐modulating adjuvants must be administered at a dosage within a clear time window. First, glutamine metabolic homeostasis is critical for T‐cell protein production and immunological activity.^163^ By antagonising Gl‐induced hydrolysis of glutamine, JHU083 treatment enhances OXPHOS in Th1 and CD8+ Teff cells to prevent apoptosis and autophagy after oxidative stress is increased, and in combination with the EGFR peptide vaccine (EVax), this treatment may promote long‐term immunity.^25^ Because Teffs lack well‐balanced effector functions, their infiltration into a setting characterised by *KRAS*, *STK11/Lkb1*, *KEAP1* and other oncogene mutant‐bearing advanced lung adenocarcinomas is severely reduced, and the rate of glutamine uptake by tumour cells is significantly increased. Notably, treatment with Gls, such as CB‐839, combined with anti‐PD‐1/PD‐L1 further reduces CD8+ T‐cell clonal expansion. Strikingly, ICB treatment requires glutamine hydrolysis, exacerbating the pressure on Teffs to utilise glutamine.^164^ Considering the multiple metabolic roles of glutamine, we speculate that there may be a precise therapeutic dosage or application window that contributes to the synergistic effect of T‐cell GIs with immunotherapy, and identifying these parameters will aid in determining the metabolic mechanisms by which glutamine positively or negatively affects tumour‐fighting immunity.

An increasing body of evidence suggests that surface proteins that distinguish the properties of different T‐cell subpopulations mediate aberrant reprogramming of T‐cell metabolism, leading to ICI resistance‐associated immunosuppressive signalling and tumour immune escape.^179^ Because CD70 expression is correlated with Treg mitochondrial wellness and intracellular lipid content, impaired CD70 expression decreases Treg adaptability to the TME, leading to loss of their effector cell‐immunosuppression support of the tumour‐escaping environment.^15^ As a consequence, patients with advanced ICI‐resistant tumours may specifically benefit from treatment combined with anti‐CD70 mAbs. Similarly, CD47 overexpression in the TME allows tumour cells to avoid immune surveillance. Blocking T‐cell CD47 expression stimulates a sustained increase in Teff glycolytic efficiency and reestablishes the classical cytotoxic phenotype.^180^ Moreover, clinical trials have demonstrated that a combination of CD47 mAbs and ICIs results in a reduced tumour burden and prolonged survival.^181^ In addition, many studies have demonstrated that blocking CD39 or CD73 inhibits extracellular ATP degradation to produce ADO,^182^, ^183^ preventing ADO from disrupting Teff cytokine production or immediate contact with cancer cells.^36^, ^179^ All of these findings suggest that remodelling T‐cell energy metabolism via anti‐surface antigen‐expressing medications may enhance the tumour immunotherapy efficacy, suggesting the existence of enduring immune responses in cancers that have acquired resistance to long‐term conventional treatments.

### 3.2 Metabolic pretreatment with CAR‐Ts may improve the immunotherapy efficacy

**FIGURE 4:**
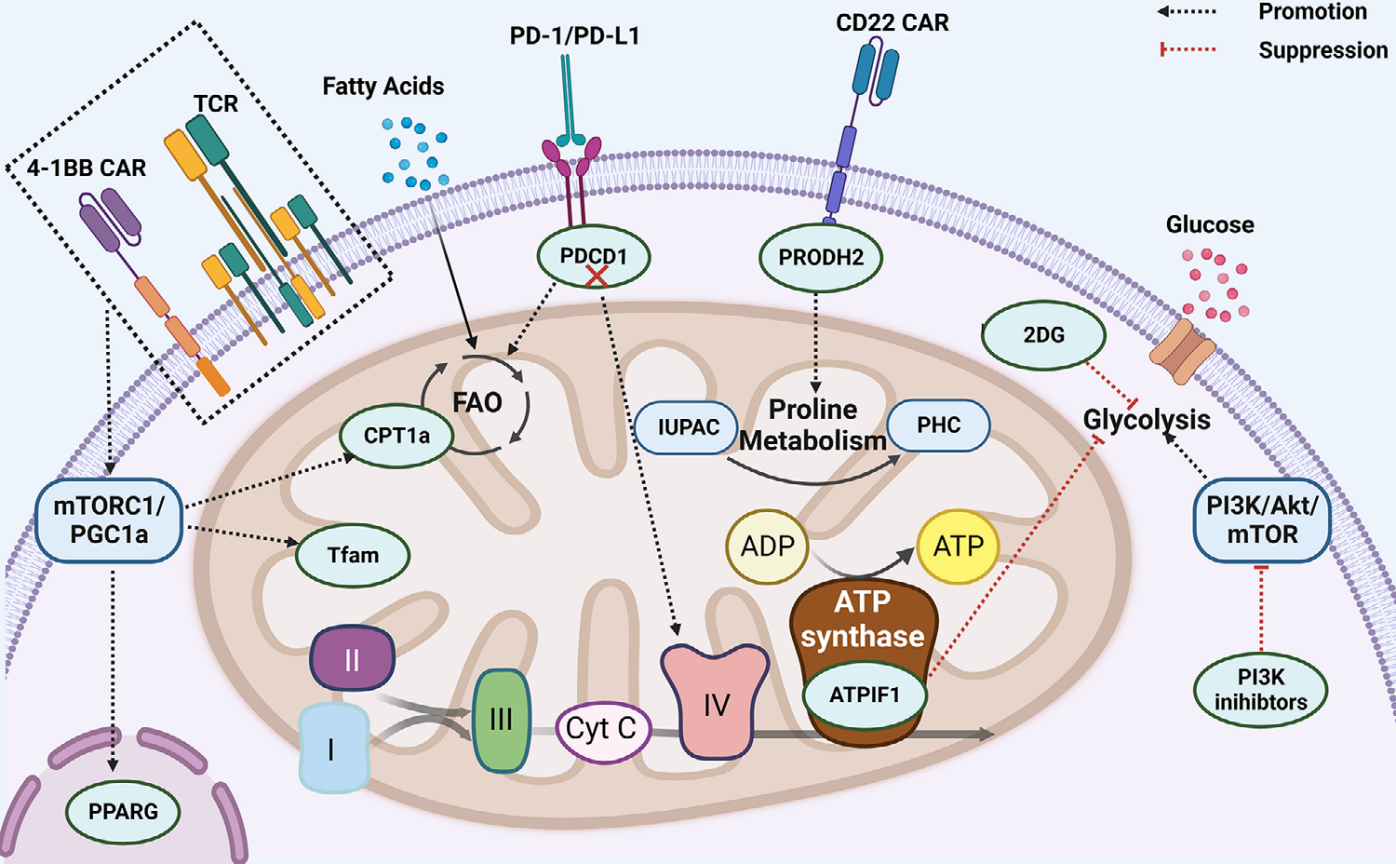
Metabolic measures and potential targets for optimising chimeric antigen receptor T‐cell (CAR‐T) therapy efficacy. CAR‐T therapy is used to target and effectively destroy tumour cells. On the one hand, the ability of the mitochondrial respiratory electron transport chain to create adenosine triphosphate (ATP) is enhanced. Furthermore, memory state differentiation is promoted by increased fatty acid and proline metabolism, and this process is necessary for the immune assault ability, lifespan and availability of T cells. Conversely, negative control of the pre‐engineered T‐cell glycolytic state inhibits T cells from adopting a glycolytic metabolism program as an alternative to mitochondrial metabolism, preventing these cells from exploiting their competitive advantage in the tumour microenvironment (TME). CAR‐Ts exert cytotoxic effects more effectively and intensively when mitochondrial function in T cells is increased and glycolytic activity in T cells is inhibited. ATPIF1: adenosine triphosphate inhibitory factor 1; IUPAC: (2S,4R)‐4‐hydroxypyrrolidine‐2‐carboxylic acid; PHC: 1‐pyrroline‐3‐hydroxy‐5‐carboxylate; PPARG: peroxisome proliferator‐activated receptor‐γ; Tfam: transcription factor A, mitochondrial.

The use of chimeric antigen receptor (CAR) in vitro to genetically alter T cells, which endows these T cells with tumour antigen‐specific effects and localised activity, has broadened the promise of tumour immunotherapy.^156^ Studies have shown that different CAR signalling domains can remodel T‐cell energy metabolism programs to mediate specific immunological differences^157^ (Figure 4). Therefore, engineered T‐cell metabolic processes may give T cells an advantage over tumour cells in competing for energy and metabolic substrates, thereby enhancing CAR‐T toxicity and durability after infusion in vivo.

Early experimental outcomes demonstrated that CAR‐Ts support robust mitochondrial fitness and enhance mitochondrial metabolism, leading to sufficient energy production for long‐lasting immunity mediated by CAR‐Ts.^184^, ^185^ CAR‐Ts with 4‐1BB/CD137 signalling exhibit a large advantage in mitochondrial oxidative activity, which increases T‐cell SRC and thus decreases the T‐cell long‐term survival energy supply.^86^, ^186^ By employing Cas9‐CRISPR editing technology to modify the expression of the PD‐1 locus in CAR‐Ts, scholars have found that the mitochondrial energy reserve capacity of 4‐1BB CAR‐Ts with PDCD1 knockdown is enhanced, which increases the OXPHOS and glycolysis rates, enabling these cells to adapt to the highly metabolic TME.^187^ Alternatively, the knocking in of proline has been found to enhance the energy reserve capacity of mitochondria. CD22 CAR‐Ts deficient in proline dehydrogenase 2 exhibit increased mitochondrial function via multiple amino acid metabolic processes to support cytolytic granule secretion and display Tm cell‐like features.^188^ In addition, activation of the mTORC1/PGC1α pathway is important for promoting mitochondrial biosynthesis, which mediates metabolic adaptability, anti‐ROS stress, and self‐renewal in CD8+ Tcm cells.^189^ The treatment of PGC1α‐overexpressing CAR‐Ts with ICI exerts a synergistic effect on enhancing the aerobic metabolic activity of T cells.^190^ The data from in vitro culture models have shown that OXPHOS and biosynthetic pathways in mitochondria may be potential targets for enhancing targeted CAR‐T antitumour immunodominance, but in vivo tumour models must also be studied to fully comprehend the mechanism of CAR‐T metabolic plasticity and viable editing.

The inhibition of CAR‐T glycolysis facilitates prolonged CAR‐T responses in the TME and prevents systemic inflammation caused by excessive cytokine release.^158^, ^191^ In contrast to CAR‐Ts, which express 4‐1BB, CD28 stimulates CAR‐Ts to transiently kill tumour cells by inducing rapid activation of core enzymes in the glycolytic pathway, transport proteins, and the PI3K/Akt signalling pathway.^157^ As large amounts of cytokines are rapidly released into the microenvironment, the risk of cytoreductive surgery greatly increases.^143^ The electron transport chain(ETC) complex V inhibitory protein adenosine triphosphate inhibitory factor 1 (ATPIF1) maintains the mitochondrial structure and the functional stability of the electron transport chain.^144^, ^192^ ATPIF1 knockdown in allogeneic CD19 CAR‐Ts increases the glycolytic activity to compensate for mitochondrial defects while decreasing the ability of these cells to generate ATP via other energy substrates, such as fatty acids or amino acids, which inhibits CD8+ Tm formation and virulence factor production and accelerates CD8+ Teff exhaustion.^159^ In summary, both the glycolysis inhibitor 2‐DG and selective PI3K inhibitors may contribute to an increased Tcm/Tem ratio and limit CAR‐T glycolytic activity to maintain persistent stemness.^160^, ^161^, ^193^ Notably, activation of the TGF‐β/Smad signalling pathway in the hypoxic TME tends to reduce T‐cell glycolytic competitiveness^194^; therefore, the knockdown of TGF‐βR2 in CAR‐Ts inhibits TGF‐β signalling to facilitate CAR‐T survival in vivo and increase the tumour elimination efficacy.^195^, ^196^, ^197^ The antitumour and anti‐self‐exhaustion activities of T cells are further increased by cotargeting CAR‐Ts with PD‐1 and TGF‐β.^198^ However, whether powerful effector or immune memory‐persistent CAR‐Ts can be used as superior weapons against tumours is debatable. In addition, the regulatory effects of TGF‐β editing on glucose metabolism and OXPHOS in CAR‐Ts and the simultaneous regulation of other cross‐talk pathways need to be further investigated.

## 4 SUMMARY AND OUTLOOK

### 4.1 Outlook

**FIGURE 5:**
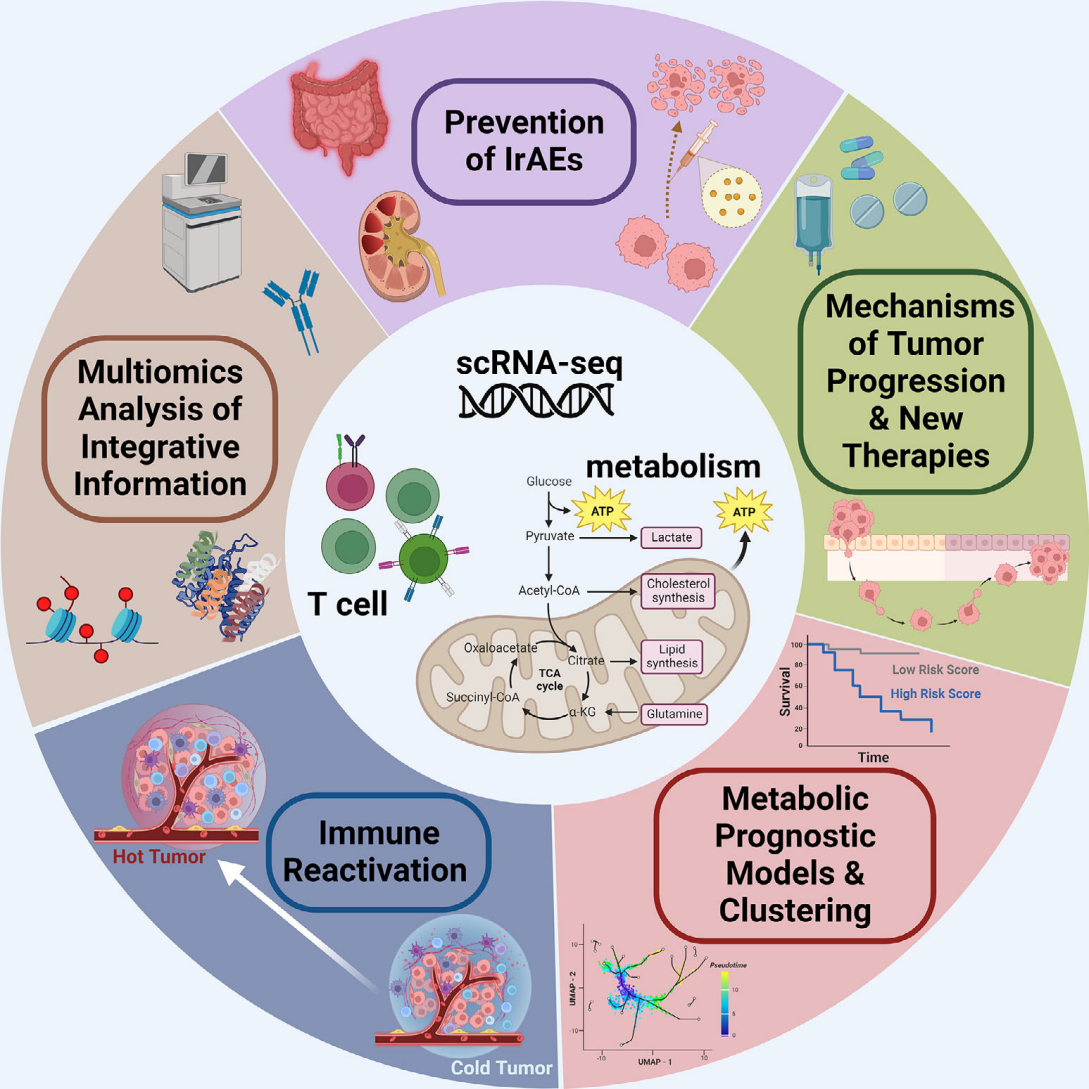
Future prospects for single‐cell transcriptome sequencing applied to T‐cell metabolism analysis. Based on the single‐cell RNA sequencing (scRNA‐seq) analysis of T‐cell metabolism, future studies on single T‐cell metabolic activity may involve the following steps: (1) designing immune reactivation therapy for immune‐desert tumours, (2) constructing metabolic prognostic models and performing clustering analysis, (3) predicting cancer survival and specifying mechanisms of tumour progression and promising therapies, (4) directing patients to an expanded realm of multiomics analysis for heterogeneous information analysis and (5) exploring the prevention of immune‐related adverse events (irAEs).

Through the maturation of scRNA‐seq‐based investigations into tumour‐specific T‐cell metabolism, the utilisation of both scRNA‐seq and T‐cell metabolic profiling data has led to continued advancements in controlling the plasticity of the TME and broadening the scope of immunotherapeutic potential. Hence, we propose several avenues for prospective and comprehensive research (Figure 5).

#### 4.1.1 Can targeted metabolism reactivate the T‐cell sensitisation response to ICB?

Although scRNA‐seq has advanced the analysis of ‘immune‐responsive’ TILs, few studies have focused on exploring the mechanisms underlying weak immune responses or the rejection of TILs moving towards the tumour margin in ‘immune‐desert’ tumours.^199^ The following question remains: Are TILs actively withdrawn or passively repelled following energy depletion? Could failed T‐cell recruitment and infiltration be attributed to environmental variants in the TME that affect T‐cell gene transcription and epigenetic characteristics? Interestingly, cold‐induced tumours are more responsive to ICIs than hot tumours, which are characterised by extensive T‐cell infiltration.^200^, ^201^ In particular, the predominant cell population within many hot tumours comprises nonreactive bystander T cells, necessitating reconsideration of the reliability of the CD8+ T‐cell abundance as a defining criterion of tumours.^202^ In summary, the multiple potential mechanisms governing the circulation and trafficking of antitumour Teffs suggest a promising niche for combination immunotherapy regimens.

#### 4.1.2 How can single‐cell multiomics overcome the limitations of using scRNA‐seq data for the analysis of T‐cell information?

Although scRNA‐seq has demonstrated powerful capabilities for identifying novel cell subpopulations and exploring cellular functional heterogeneity, single‐cell proteomics (sc‐proteomics), which analyses proteins as functional molecules translated and modified from transcribed RNA, offers several advantages. First, solely depending on RNA abundance cannot directly infer protein abundance within cells, and the results are limited to the transcriptome level. Second, proteomic analysis at single‐cell resolution can reveal posttranslational modifications that are not achievable via transcriptomics. Third, sc‐proteomics enables multilevel analysis of cell crosstalk through the expression of proteins involved in intercellular ligand–receptor interactions, adhesion molecules, enzymes and other proteins. This perspective allows the interpretation of complex tumour immune microenvironments from the viewpoint of intercellular interactions.^203^, ^204^ In summary, the heterogeneity of the numerous T‐cell populations in the TME, which varies depending on their differentiation states, metabolic patterns and functional effects, is often not limited to or is completely independent of transcriptome differences and thus cannot be fully captured solely through scRNA‐seq data.^205^, ^206^, ^207^ Therefore, the emergence of single‐cell multiomics technologies is highly important for addressing these challenges. Investigating single‐cell chromosome accessibility,^208^ tissue spatial mapping, membrane protein expression profiling and TCR‐seq will lead to comprehensive characterisation of individual T‐cell molecules and locations, multiple signatures of linked gene and protein expression, and immunopharmaceutical target screening.^209^, ^210^

For instance, the functional state heterogeneity, metabolic plasticity and specific responses to ICIs mediated by γδT cells remain unknown. Distinguishing γδT‐cell subpopulations based on developmental sites, structures or effector molecules has failed to enable sufficient identification of overlapping classifications or elucidation of changes in specific metabolic and genomic chromatin states in response to dysfunction. We envision that the expression of γδT‐cell‐specific markers, epigenetic changes in response to PAg stimulation and associated metabolic remodelling processes, interactions of γδT cells with αβT cells in the TME, their relative positions, and combined drug‐induced increases in the abundance of these molecules can be revealed through the use of single‐cell transcriptomic and multiomics research techniques to develop feasible targeted therapeutic regimens based on γδT cells in the future.^138^, ^211^

Moreover, metabolomics analysis, which encompasses metabolic gene expression levels, regulatory behaviours, substrate binding, metabolic enzymes and correlations among generated metabolites, is increasingly being applied in single‐cell research.^212^ By applying single‐cell multiomics analysis to the development of CAR‐T therapy, researchers can intensively investigate cell genomes, spatial relationships among cells, and other critical information by continuously tracking dynamic changes in T‐cell subpopulation clones via measurements of differentiation and mutation markers and intercellular interaction pathway activation levels. This approach will lead to the identification of key molecules for improved CAR‐T therapy that are very precise, the assessment of the impact of mitochondrial metabolism levels and the TME on the functional status of CAR‐Ts after infusion from multiple angles, and the prediction of the degree of clinical benefit achieved with CAR‐T therapy.^189^

Because previous studies mainly focused on single‐cell transcriptomics, the analysis of complex cellular metabolic patterns should fully consider the interplay among individual cells, various metabolites, functional small molecules and the characteristics of cell epigenetics. However, these aspects represent a significant unexplored territory. In summary, future investigations should additionally employ multiomics integrative research methods to explore in depth the actual immune status and metabolic patterns of clinical diseases, thereby providing additional convincing evidence for the association between T‐cell metabolism and immune function and establishing metabolism‐related predictive models for different tumour treatment strategies.

#### 4.1.3 Can the metabolic patterns of individual T cells reveal new targets for future survival prognostic probes?

Indeed, integrating the T‐cell metabolome with genome biomarker analyses to classify endogenous metabolic pathways may add a new dimension to prognostic predictions of tumour therapy.^11^, ^213^ Recently, a lipid metabolism gene prognostic signature was successfully validated for tumour patient survival, immune infiltration, cell mutation and treatment prognosis^214^, ^215^ and was also validated via scRNA‐seq‐based construct analysis for patients with hepatocellular carcinoma.^216^, ^217^ Furthermore, the effects of several metabolic pathways that have received less attention, such as selenium metabolism,^218^ polyamine metabolism^219^ and folate metabolism,^220^ on tumour prognosis have been supported by preliminary validation data. In the context of immunotherapy, the innovative value of several methods, including the T‐cell‐related prognostic index and the multimetric analysis of biomarkers for immunotherapy, such as CAMOIP, for assessing the prognosis of patients treated with ICIs has been identified as an additional direction for the development of new prognostic models made possible by immunometabolomics.^221^, ^222^ In addition, scRNA‐seq has proven valuable for mining T‐cell marker genes of lung adenocarcinoma,^223^ lung squamous cell carcinoma^224^ and triple‐negative breast cancer^225^ and identifying the associated enrichment terms and pathways to construct independent predictive models of disease risk and immunotherapy responses. The comprehensive coverage of differentially expressed genes, the robust analysis of gene set enrichment pathways, and the sensitive and specific detection of tumour infiltration‐related genes in T cells make single‐cell‐based sequencing data a valuable resource for constructing precise and sensitive infiltrating T‐cell metabolic prognostic risk models.^226^

#### 4.1.4 Can complex interactions among cells involved in different metabolic processes in the TME be characterised to elucidate the molecular mechanisms underlying tumour progression or the therapeutic potential of adaptive T cells?

We speculate that the relationship between the antitumour immune response of T cells and pericyte activity may be mediated through intercellular communication pathways or may be related to increased metabolic waste accumulation within pericytes. For example, mitochondrial dysfunction in hepatocellular carcinoma cells after Crif knockout induces increased glucose catabolism to pyruvate and lactate overaccumulation. The unfavourable cellular environment caused by this process leads to downregulated expression of surface effector receptors (IFN‐γ, tumour necrosis factor [TNF] and T‐bet) in infiltrating Teffs.^109^ Metabolism‐related interactions between malignant cells and T cells play critical roles in shaping the immune response within the TME, providing fresh insights into strategies for targeting tumour cell metabolism to mediate and improve T‐cell metabolism remodelling.

In addition, novel tumour therapeutic agents (e.g., cuproptosis and DNA damage response inhibitors) are thought to recruit IFN‐γ‐, TNF‐α‐ and IL‐2‐secreting T cells to the TME by enhancing the activation of the cGAS–STING–IFN intrinsic immune pathway, which acts as an intermediary for T‐cell recruitment and remodelling of the immune‐suppressive metabolic state of tumour cells, such as IDO‐1‐mediated tryptophan degradation and dysregulated fatty acid metabolism.^227^, ^228^, ^229^ Notably, intrinsic‐like T cells, such as CD161^hi^+CD8+ T cells, often fail to recognise tumour subclone antigens and exhibit limited tumour lysis and clonal expansion capacity in the context of recurrent/metastatic tumours.^230^, ^231^, ^232^ Additionally, dying processes, such as epithelial–mesenchymal transition and NOTCH signalling, are often significantly associated with increases in aerobic metabolism and active exhausted T‐cell interaction pairs in tumours with a negative prognosis.^233^, ^234^, ^235^ Clearly, elucidating the accelerated T‐cell exhaustion process without enhancing the intrinsic killing effect is key for developing novel therapies to benefit primary and recurrent/metastatic patients.

#### 4.1.5 Can reprogramming T cells and microbial metabolic processes effectively reduce the number of irAEs?

Several single‐cell studies have indicated that ILTC metabolism tends to shift towards FAO, which inhibits the release of the inflammatory regulator IL‐22, an initiating factor that increases the development of colitis following anti‐PD‐1 therapy.^236^ Unexpectedly, combining metabolic modulators with ICI treatment may increase the incidence of irAEs.^176^ This outcome underscores the need for rational solutions to overcome irAEs caused by ICIs by blocking the abnormal metabolic reprogramming of T cells or actively directing the T‐cell metabolic process. Modifying the intestinal microbial system and thus enhancing immunotherapy often relies on short‐chain fatty acids to ensure tumour cell histone acetylation, which creates a favourable environment for beneficial microorganisms and the antitumour immune system. This method may prevent the occurrence of irAEs in the digestive system to some extent.^237^, ^238^, ^239^ Currently, we emphasise the need for thorough in vitro validation using multiple experimental cell line models together with in vivo experiments using animal models and human participants to gain insights into the mechanisms that are co‐manipulated by drug combinations.

## 5 CONCLUSIONS

Scientists have focused on the use of flow cytometry analysis to study the differences between T‐cell subpopulations since the early days. Over the past decade, the transitional or masked differentiation phenotypes of T cells after activation have begun to be identified at the single‐cell transcriptional level with increasing precision, thus revealing the heterogeneous characteristics of T‐cell subpopulations that have been overlooked. Concurrently, the accuracy can be validated with downstream experiments such as flow cytometry. However, we must acknowledge that over‐segmentation of cell populations remains a risk with scRNA‐seq, which could conflate subpopulations of true biological significance from hypothetical ones. Additionally, scRNA‐seq advanced computational methods, such as dynamical trajectory modelling, differential expression or pathway enrichment analysis and dimensionality reduction, to deeply profile cellular heterogeneity and multi‐dimensional states. Overall, integrating scRNA‐seq with complementary experimental strategies holds promise to advance our understanding of T‐cell diversity, plasticity and function at single‐cell resolution.

Through antitumour immunity, metabolism in different T‐cell subpopulations is regulated by surrounding factors, such as changes in the surrounding environment, metabolic enzyme activities and signals delivered by antigenic stimuli, which provide specific instructions to the immune machinery in cell subpopulations. Therefore, the dynamics of metabolism, convergence, and heterogeneity of both TILs and peripherally circulating reactive T‐cell subpopulations determine T‐cell survival, cytotoxicity, proliferation and growth‐related signalling pathway activity; intracellular amino acid or protein abundance; and the direction of differentiation and phenotype acquisition. This complexity implies that understanding the relevant mechanisms by which the different metabolic programs of T cells govern immune functions and integrate metabolic patterns and signalling factors that link or constrain T‐cell subpopulations to the TME will enable further expansion of tumour therapeutic strategies in the field of immunometabolism.

## AUTHOR CONTRIBUTIONS

Jian Zhang, Peng Luo and Ting Wei conceived the study and provided constructive guidance. Lihaoyun Huang and Haitao Li collected and organised the related references and prepared all the figures. Lihaoyun Huang wrote the manuscript. Lihaoyun Huang and Haitao Li corrected the manuscript. Lihaoyun Huang, Haitao Li, Cangang Zhang, Quan Chen and Zaoqu Liu made significant revisions to the manuscript. Lihaoyun Huang and Haitao Li prepared the table and managed the data. Cangang Zhang, Quan Chen and Zaoqu Liu perfected the manuscript. Lihaoyun Huang and Haitao Li edited the manuscript for proper use of the English language to obtain the final draft. All the authors read and approved the final version of the manuscript.

## CONFLICT OF INTEREST STATEMENT

The authors declare they have no conflicts of interest.

## ETHICS STATEMENT

Not applicable.

## CONSENT FOR PUBLICATION

The authors agree to the publication of all the data presented in this article. No data from other entities were used in this study.

## Data Availability

Not applicable.
